# Perceived Confucian Ren-Based Teaching: scale development, construct validation, and associations with learning motivation and school belonging

**DOI:** 10.3389/fpsyg.2026.1898379

**Published:** 2026-09-30

**Authors:** Wei Lu

**Affiliations:** College of Literature, Hubei University of Education, Wuhan, Hubei, China

**Keywords:** benevolent teaching, Confucianism, ethic of care, learning motivation, ren (benevolence), school belonging, teacher–student relationship

## Abstract

**Introduction:**

Teacher care is closely linked to students' motivation and sense of belonging, yet commonly used teacher-care measures have been developed largely within Western educational psychology and the ethics of care. Confucian ren offers a culturally grounded account of humane teaching but has rarely been operationalized as a student-perceived classroom construct.

**Methods:**

Across two independent convenience samples of post-secondary students recruited from three institutions in Hubei Province, China, we developed and validated the Perceived Confucian Ren-Based Teaching (PRBT) scale and examined its associations with learning motivation and school belonging. Study 1 (*N* = 326) used expert review, cognitive interviews, parallel analysis, and exploratory factor analysis. Study 2 (*N* = 512) used confirmatory factor analysis and structural equation modeling to examine measurement quality, associations with an abbreviated care-oriented teaching benchmark, and theoretically ordered indirect associations through teacher-student relationship quality and basic psychological need satisfaction.

**Results:**

Study 1 supported a 24-item, six-factor structure comprising benevolent concern, inclusive teaching, patient guidance, responsive instruction, norm-guided care, and moral modeling. In Study 2, a correlated six-factor model fit well and retained the clearest dimension-level interpretation, while a second-order model also supported qualified use of a total composite. PRBT was strongly associated with, but empirically distinct from, the pre-specified five-item abbreviated care-oriented teaching benchmark. The theoretically ordered serial model had the lowest AIC and BIC, although a parallel specification showed marginally better comparative fit index (CFI) and standardized root mean square residual (SRMR). PRBT also explained additional variance in learning motivation and school belonging after demographic covariates and the abbreviated care-oriented teaching benchmark were controlled.

**Discussion:**

The findings provide initial support for PRBT as a culturally grounded, multidimensional operationalization of humane teaching and for cross-sectional indirect associations consistent with the proposed relational-motivational ordering. Because all focal variables were measured by student self-report on one occasion, these associations should not be interpreted as a causal mediation sequence. Replication with full-length comparison measures, multi-wave and multi-source designs, and samples from other regions and educational contexts are needed.

## 1 Introduction

The quality of the relationship a student has with a teacher is one of the most consistent relational correlates of motivation, engagement, and belonging in educational settings. Meta-analytic evidence links affectively positive, supportive teacher–student relationships to higher engagement and achievement across grade levels (Roorda et al., 2011), and studies of teacher support and pedagogical caring show that students who experience their teachers as caring report stronger academic motivation, more prosocial classroom goals, and a greater sense of relatedness (Wentzel, 1997, 1998; Furrer and Skinner, 2003). Within educational psychology these effects have most often been framed through the ethics of care, especially Noddings's account of caring (Noddings, 1984, 2005) as receptive attention and motivational displacement toward the cared-for, and through self-determination theory, which explains why relational warmth matters by tying it to the satisfaction of basic psychological needs (Ryan and Deci, 2000; Niemiec and Ryan, 2009). Both frameworks were elaborated largely in Western educational settings, and the instruments that operationalize teacher care–perceived caring, immediacy, and teacher support–reflect that provenance.

Yet many students learn in Confucian-heritage educational cultures whose image of the good teacher is also articulated through a different moral vocabulary. In that tradition the central virtue is *ren*, the relational excellence that Confucius, asked directly what it is, glossed simply as “loving others” (*Analects* 12.22) (Confucius, 1979, 2003). As applied to teaching, *ren* supports a recognizable ideal: a teacher who cares for students as whole persons, teaches without class distinctions (*Analects* 15.39), instructs without weariness (7.2), responds to differences among learners, upholds shared norms, and teaches through personal moral example (13.6; 14.42) (Confucius, 1979, 1971, 1998, 2003). Importantly, the present argument does not treat benevolence, patience, or responsiveness as behaviors unique to Confucian teaching. These qualities are also central to established teacher-care and teacher-support constructs. The proposed distinctiveness of Perceived Confucian Ren-Based Teaching (PRBT) is instead configurational: relational concern is organized together with inclusive obligation, norm-guided benevolence, moral exemplarity, and a role-structured orientation toward students' broader cultivation. Thus, PRBT is expected to overlap substantially with teacher caring while representing a somewhat different normative organization of humane teaching, rather than a culturally exclusive set of behaviors (Markus and Kitayama, 1991; Tweed and Lehman, 2002; Li, 2012).

This gap has two costs. Empirically, relying on a single care-oriented operationalization in Confucian-heritage settings may underrepresent culturally salient features of humane teaching if those features include role obligation, norm-guided warmth, and moral exemplarity in addition to responsiveness. Theoretically, the field lacks evidence on whether a culturally grounded model of benevolent teaching is associated with outcomes such as motivation and belonging, and whether it accounts for additional variance relative to an established care-oriented benchmark. A philosophical comparison of *ren* and care, which a sizeable literature has pursued (Li, 1994; Star, 2002; Tao, 2000), cannot by itself settle these measurement and nomological questions; they require an explicit construct definition and empirical evaluation.

The present research addresses the gap directly. We develop a student-rated Perceived Confucian Ren-Based Teaching scale that is conceptually grounded in specific passages of the *Analects* and in contemporary pedagogical interpretations of those passages, and we situate it within a relational–motivational model informed by self-determination theory. We then make three contributions. First, we evaluate whether ren-based teaching can be measured as a coherent, reliable, multidimensional construct and whether it is positively associated with learning motivation and school belonging (H1 and H2), using exploratory and confirmatory factor analysis and structural equation modeling. Second, we test whether the observed cross-sectional associations are statistically consistent with a theoretically ordered pathway in which PRBT is associated with teacher–student relationship quality, which is associated with basic psychological need satisfaction, which is in turn associated with motivation and belonging (H3 and H4). Bootstrap indirect-association estimates and competing structural specifications are used to evaluate this pattern, but not to infer temporal mediation. Third, for the cross-cultural measurement argument, we test whether PRBT is empirically distinguishable from, while substantially overlapping with, a care-oriented teaching benchmark (H5), and whether it accounts for additional outcome variance after demographic covariates and that benchmark are entered (H6). Because the comparison measure is an abbreviated five-item form, H5 and H6 speak specifically to distinctiveness and incremental association relative to that operationalization rather than to the entire domain of teacher caring.

## 2 Theoretical background and hypotheses

### 2.1 Ren-based teaching: six dimensions conceptually grounded in the *Analects*

We define *perceived ren-based teaching* as students' perception that their teacher enacts, in the classroom, a pattern of humane teaching organized around the Confucian relational virtue of *ren*. The wording “grounded in the *Analects*” denotes conceptual grounding in identifiable passages rather than a claim that every contemporary dimension label, or every item-level formulation, appears verbatim in the classical text. To make the source status explicit, we distinguish three forms of grounding: (a) education-specific textual anchors in passages explicitly concerning teaching, (b) pedagogical applications of broader Confucian principles to the teacher role, and (c) interpretive synthesis across passages whose educational implication is learner-sensitive guidance. Multiple English translations were consulted to reduce dependence on any single rendering (Confucius, 1979, 1971, 1998, 2003).

Six dimensions were distinguished, with different source-status claims. *Benevolent concern* takes “loving others” (*ai ren*; 12.22) as a broad *ren* principle and applies it pedagogically to care for students as persons rather than only as bearers of grades. *Inclusive teaching* is anchored more directly in the educational formulation *you jiao wu lei* (15.39), the commitment to teach without class distinctions. *Patient guidance* similarly draws on the education-specific formulation of teaching “without weariness” (7.2), operationalized as persistent explanation and guidance when students have difficulty. *Responsive instruction* denotes differentiated, learner-sensitive guidance. The later four-character formulation *yin cai shi jiao* is not a verbatim *Analects* phrase; this dimension is therefore treated as an interpretive synthesis of passages in which Confucius gives different answers to Zilu and Ran You in view of their dispositions (11.22) and distinguishes what may be taught to learners at different levels (6.21). *Norm-guided care* applies the broader relation between *ren* and ritual propriety in 12.1 to a pedagogical pattern of warmth exercised within clear and fair expectations. *Moral modeling* is likewise a pedagogical application of broader Confucian claims about self-cultivation and exemplary conduct, drawing on 12.1, 13.6, and 14.42. These distinctions prevent the scale from treating contemporary psychological labels as literal classical categories.

Table 1 summarizes both the conceptual content and the grounding status of each dimension. The closest recent work is a Chinese scale of teacher caring behavior in online teaching (Zhao et al., 2023); PRBT differs not because every component behavior is uniquely Confucian, but because it organizes benevolent, inclusive, patient, differentiated, norm-guided, and exemplary facets into a single role-structured account of humane teaching in a general classroom context. This framing deliberately avoids treating the six English labels as canonical expressions.

**Table 1:** Conceptual sources and item map for the Perceived Confucian Ren-Based Teaching scale.

Dimension	Educational meaning	Classical source	Grounding status	Example item
Benevolent concern	Caring for students' growth and wellbeing	*Analects* 12.22: Fan Chi asked about *ren*; the Master said, “love others.”	Pedagogical application of a broad *ren* principle	My teacher cares about students' overall growth, not only grades.
Inclusive teaching	Fair and inclusive treatment	*Analects* 15.39: in teaching, there should be no class distinctions.	Education-specific textual anchor	My teacher gives equal learning opportunities to students with different backgrounds.
Patient guidance	Patient and persistent guidance	*Analects* 7.2: learning without satiety and teaching without weariness.	Education-specific textual anchor	My teacher explains patiently when students have difficulty understanding.
Responsive instruction	Differentiated responses to students	*Analects* 11.22 and 6.21: differentiated responses are inferred from attention to learners' dispositions and levels.	Interpretive synthesis across passages	My teacher adjusts guidance according to different students' learning needs.
Norm-guided care	Care with boundaries and norms	*Analects* 12.1: to restrain oneself and return to ritual propriety is *ren*.	Pedagogical application of a broader normative principle	My teacher's care has clear principles rather than simply indulging students.
Moral modeling	Influence through moral example	*Analects* 12.1, 13.6, and 14.42: self-cultivation and exemplary conduct orient one's influence on others.	Pedagogical application of broader self-cultivation and exemplary-conduct principles	My teacher influences students through consistent moral example.

### 2.2 Teacher caring and the ethic of care in education

A prominent comparison framework in educational psychology is teacher caring as theorized in the ethics of care, the family of relationship-centered moral theories developed by Gilligan (1982), Noddings (1984), Held (2006), and Slote (2007), among others. Noddings (1984, 2005) gave the position its most influential statement for education, locating the moral relation of teaching in the encounter between the “one-caring” and the “cared-for”: the caring teacher attends receptively to the student and is moved to take up the student's ends as her own. Empirical work in this tradition shows that perceived caring and teacher support relate to academic motivation, classroom engagement, and prosocial goal pursuit (Wentzel, 1997, 1998).

Ren-based and care-oriented teaching plainly overlap: both are relational, affectively engaged, and attentive to the concrete student rather than governed only by detached rule. Benevolent concern, patient guidance, and responsive instruction therefore should not be interpreted as uniquely Confucian behaviors; close analogs appear in teacher caring, teacher support, and need-supportive teaching. The conceptual distinction proposed here lies at the level of how these behaviors are organized and what normative work they are expected to perform. In PRBT, concern and responsiveness are embedded within a role-structured teacher–student relationship that also treats inclusion as an obligation, links warmth to fair normative expectations, and gives the teacher's own moral conduct an explicitly exemplary function. The teacher is therefore not only a responsive relational partner but also a role bearer whose conduct is oriented toward students' broader cultivation.

This distinction should not be overstated. The ethics-of-care literature is internally diverse, and teacher-care constructs can include fairness, guidance, and developmental concern. Our claim is consequently not that PRBT contains content wholly absent from all Western theories of caring. Rather, PRBT makes a particular configuration of relational concern, inclusive obligation, norm-guided benevolence, and moral exemplarity constitutive of the construct and anchors that configuration in Confucian textual and educational sources. Empirically, the appropriate expectation is substantial overlap with care-oriented teaching combined with discriminant validity. Any incremental validity finding must also be interpreted relative to the specific care measure used in this study, not as evidence that PRBT supersedes the broader construct of teacher caring.

### 2.3 An SDT-informed relational–motivational model: relationships, needs, motivation, and belonging

Self-determination theory (SDT) offers a theoretical account of why relationally warm teaching may be associated with adaptive student outcomes (Deci and Ryan, 1985; Ryan and Deci, 2000, 2017). SDT holds that motivation and wellbeing depend on the satisfaction of three basic psychological needs–autonomy, competence, and relatedness–and that social contexts foster autonomous motivation to the extent that they support these needs (Chen et al., 2015). In educational settings, need-supportive teaching predicts more autonomous forms of academic motivation and adaptive outcomes (Niemiec and Ryan, 2009; Deci et al., 1991).

The proposed ordering moves from a perceived feature of the teaching environment to a relationship-specific appraisal and then to a more proximal psychological experience. PRBT describes what students perceive the teacher to do across recurring classroom encounters. Those encounters provide cues from which students form an appraisal of the particular teacher–student relationship, including trust, satisfaction, instrumental help, and conflict (Ang, 2005; Ang et al., 2020). A relationship experienced as dependable and low in conflict can then function as the immediate interpersonal context in which autonomy, competence, and relatedness are supported or frustrated (Furrer and Skinner, 2003; Chen et al., 2015). Need satisfaction is consequently positioned closer to motivation and belonging than the broader relationship appraisal.

This ordering is theoretically plausible but is not mandated uniquely by SDT. Need satisfaction may shape subsequent perceptions of relationship quality, and relationship quality and need satisfaction may operate partly in parallel or reciprocally. We therefore treat the serial chain as a theoretically ordered associational model and compare it with parallel and reverse-chain alternatives. The cross-sectional design cannot establish temporal precedence even when one model has more favorable information criteria.

### 2.4 The present research and hypotheses

The preceding review suggests that ren-based teaching and care-oriented teaching share an important relational foundation but organize that foundation differently. PRBT does not claim cultural ownership over benevolence, patience, or responsiveness. Its proposed added content lies in the integration of those relational qualities with inclusive obligation, moral exemplarity, norm-guided benevolence, and a role-structured orientation toward students' cultivation. This conceptual overlap and difference create two empirical questions. First, can perceived ren-based teaching be measured as a coherent multidimensional classroom construct grounded in Confucian educational thought? Second, does this culturally grounded construct account for additional variance in students' motivation and belonging relative to the abbreviated care-oriented teaching benchmark used in the present study?

To address these questions, the present research combines scale development with hypothesis testing. Study 1 examines the latent structure of the newly developed Perceived Confucian Ren-Based Teaching scale and evaluates whether the theoretically proposed dimensions are recoverable in students' responses. Study 2 confirms the measurement structure in an independent sample and tests cross-sectional associations within a theoretically ordered relational–motivational model. Students who perceive their teacher as benevolent, inclusive, patient, responsive, norm-guided, and morally exemplary are expected to report higher-quality teacher–student relationships; relationship quality is expected to covary positively with basic psychological need satisfaction; and need satisfaction is expected to covary positively with learning motivation and school belonging. These expectations concern indirect association patterns in same-occasion data and do not require the assumption that the proposed variables changed in that temporal order.

Learning motivation and school belonging were selected as the primary outcomes because they represent two central ways in which humane teaching may matter for students: motivation reflects students' willingness to invest effort in learning, whereas belonging reflects their sense of being accepted and included within the school community. Academic engagement was retained as an a priori secondary criterion of nomological breadth rather than as a third focal outcome. It captures energetic and behaviorally expressed involvement and was analyzed separately so that it did not enlarge the primary hypothesis set. We therefore asked an exploratory question: does PRBT show incremental association with academic engagement after demographic covariates and the abbreviated care-oriented teaching benchmark are entered? The comparison with care-oriented teaching is central to the cross-cultural contribution of the study. If PRBT merely repackages the content represented by this benchmark, it should show little empirical distinctiveness or incremental association. If it captures additional variance related to its broader Confucian configuration, it should correlate substantially with the benchmark while remaining empirically discriminable and account for additional variance in student outcomes. Because the benchmark is abbreviated, this test is deliberately narrower than a claim of incremental validity over the full theoretical domain of teacher caring.

On this basis, we tested six hypotheses:

- H1. Perceived ren-based teaching is positively associated with students' learning motivation.
- H2. Perceived ren-based teaching is positively associated with students' school belonging.
- H3. Perceived ren-based teaching shows positive indirect associations with learning motivation and school belonging through teacher–student relationship quality.
- H4. Perceived ren-based teaching shows positive serial indirect associations with the outcomes through teacher–student relationship quality and basic psychological need satisfaction, consistent with the theoretically ordered pattern PRBT → relationship quality → need satisfaction → outcomes.
- H5. Perceived ren-based teaching is substantially and positively correlated with the abbreviated care-oriented teaching benchmark but is empirically distinct from it.
- H6. Perceived ren-based teaching accounts for additional variance in learning motivation and school belonging after demographic covariates and the abbreviated care-oriented teaching benchmark are entered.

Together, these hypotheses allow the study to evaluate ren-based teaching at three levels: its internal measurement structure, its cross-sectional relational–motivational association pattern, and its incremental association relative to the abbreviated care-oriented teaching benchmark. The separate academic-engagement analysis addresses the exploratory question and is interpreted as supportive rather than confirmatory evidence. Figure 1 presents the hypothesized ordered indirect-association model. Because all focal variables were measured on the same occasion, the arrows represent theoretical ordering for statistical evaluation rather than observed temporal succession or causal effects.

**Figure 1:**
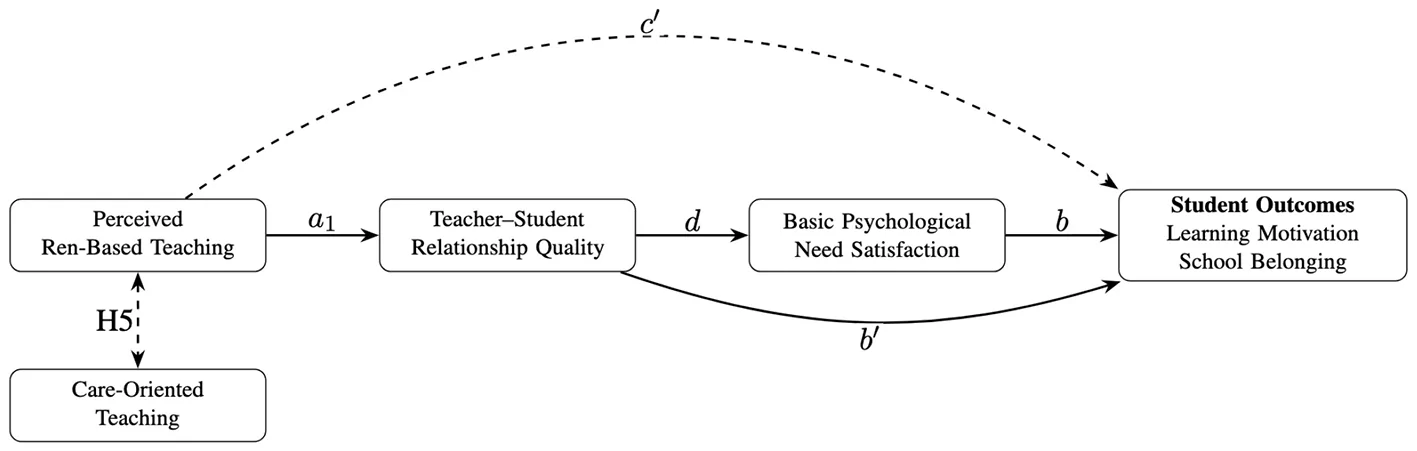
Hypothesized theoretically ordered indirect-association model. Perceived ren-based teaching is expected to be associated with student outcomes both directly and indirectly through teacher–student relationship quality and basic psychological need satisfaction. The dashed curved path denotes the residual direct association after the intermediate variables are included, and the dashed double-headed arrow denotes the conceptual and empirical comparison between ren-based teaching and care-oriented teaching. Solid arrows represent focal theoretically ordered associations. Because the design is cross-sectional, the arrows do not establish temporal succession or causal effects.

## 3 Materials and methods

### 3.1 Participants and procedure

The instrument was developed and validated with two independent convenience samples of post-secondary students.

Participants were recruited from three higher education institutions in Hubei Province, China: Jianghan University, Hubei University of Education, and Hubei Urban Construction Vocational and Technical College. These institutions represented a comprehensive university, a normal university, and a vocational college, respectively. Convenience sampling was used. Students enrolled in selected courses were invited to complete the survey, and participation was entirely voluntary.

Study 1 was conducted from October to November 2025 and Study 2 from March to April 2026. The same three institution types were represented in both studies, but Study 2 was recruited from course sections that had not participated in Study 1, preventing participant overlap.

Student-affairs staff and course coordinators distributed a neutral invitation containing a QR code and survey link. Instructors were not present when students decided whether to participate and did not receive individual responses. Eligibility required being at least 18 years old, currently enrolled at one of the participating institutions, and having completed at least 4 weeks of a recent course taught by the teacher being rated. Participants were instructed to identify one clearly specified recent course before answering all teacher-referent items. Participation was voluntary, anonymous, and uncompensated. Electronic informed consent appeared before the questionnaire and students could exit without submitting.

Data-screening rules were specified before the focal analyses and applied sequentially so that exclusion counts were mutually exclusive. Cases were excluded for more than 20% missing substantive responses; failure on either of two instructed-response attention checks; a long-string pattern in which at least 80% of substantive Likert items received the same response; or completion time below one-third of the sample median (Study 1: <6.2 min; Study 2: <6.0 min). In Study 1, 382 students opened the survey, 361 consented, and 352 submitted questionnaires. Sequential exclusions for missingness (*n* = 8), attention-check failure (*n* = 7), long-string responding (*n* = 6), and short duration (*n* = 5) yielded *N* = 326 (92.6% of submitted questionnaires). In Study 2, 604 students opened the survey, 575 consented, and 558 submitted questionnaires. Sequential exclusions for missingness (*n* = 14), attention-check failure (*n* = 13), long-string responding (*n* = 11), and short duration (*n* = 8) yielded *N* = 512 (91.8% of submitted questionnaires). Item-level missingness after exclusions was 0.8% in Study 1 and 0.6% in Study 2. Full-information maximum likelihood (FIML) was used for CFA and SEM. Absolute skewness was below 2 and kurtosis below 7 for retained indicators. Table 2 reports sample characteristics and the distribution across institution types.

**Table 2:** Sample characteristics for Study 1 and Study 2.

Category	Level	Study 1 (*N* = 326)	Study 2 (*N* = 512)
Sample size	Total analytical sample	326	512
Gender	Female	178 (54.6%)	286 (55.9%)
Gender	Male	148 (45.4%)	226 (44.1%)
Grade	First year	88 (27.0%)	130 (25.4%)
Grade	Second year	91 (27.9%)	138 (27.0%)
Grade	Third year	83 (25.5%)	129 (25.2%)
Grade	Fourth year	64 (19.6%)	115 (22.5%)
Age	Mean (SD), years	20.12 (1.31)	20.18 (1.37)
Family SES	Mean (SD), 1–5 scale	3.09 (0.78)	3.13 (0.81)
Prior achievement	Self-reported GPA percentile, mean (SD)	67.4 (15.2)	68.1 (15.7)
Institution	Jianghan University (comprehensive)	194 (59.5%)	311 (60.7%)
Institution	Hubei University of Education (normal)	80 (24.5%)	126 (24.6%)
Institution	Hubei Urban Construction Vocational and Technical College (vocational)	52 (16.0%)	75 (14.6%)

The achieved sample sizes exceeded the design targets in the accompanying power analysis, following established guidance for covariance-structure power, mediated effects, and regression power (MacCallum et al., 1996; Fritz and MacKinnon, 2007; Faul et al., 2009). For Study 1 EFA, the available *N* = 326 exceeded the minimum targets of 252 cases for item-pool screening and 300 cases for stable factor recovery. For Study 2 CFA/SEM, the available *N* = 512 exceeded the RMSEA-based SEM target of 403 cases, the bootstrap indirect-effect target of 400 cases, the incremental-regression target of 395 cases, and the measurement-invariance targets for gender and grade group analyses.

### 3.2 Measures

Unless otherwise noted, items were rated on a 5-point scale (1 = *strongly disagree* to 5 = *strongly agree*). Established English-language measures were translated by one bilingual educational-psychology researcher and independently back-translated by a second translator who had not seen the originals. A panel of two educational psychologists and one bilingual higher-education researcher resolved discrepancies. Twelve students then completed cognitive interviews focused on comprehension, referent consistency, and culturally unnatural wording; minor wording changes did not alter item meaning.

#### 3.2.1 Perceived Confucian ren-based teaching

Thirty-six candidate items (six per proposed dimension) were generated from the conceptual map. Seven experts–two in Confucian philosophy, two in educational psychology, two in higher-education pedagogy, and one in psychometrics–rated each item for relevance, clarity, and fidelity to its stated source on a four-point scale. Item-level content-validity indices ranged from 0.86 to 1.00 and the scale-level average was 0.95; eleven items were reworded before field administration, but all 36 were retained for empirical screening. The expert instructions distinguished dimensions with education-specific textual anchors from pedagogical applications of broader Confucian principles and from interpretive synthesis. In particular, responsive instruction was explicitly described as an interpretive synthesis of *Analects* 11.22 and 6.21, not as a verbatim classical formulation. Study 1 retained 24 items, four per dimension. No retained item is reverse scored. Dimension scores are means of their four items; the total composite is the mean of the six dimension scores when at least five dimensions are available. The complete Chinese and English wording and retention decisions for all 36 fielded items are reported in Supplementary Table S1. The full Study 1 cr oss-loading matrix and communalities, together with the Study 2 standardized CFA loadings for the retained 24 items, are reported in Supplementary Table S2.

#### 3.2.2 Care-oriented teaching

The comparison construct was measured with a pre-specified five-item abbreviated form of the 10-item bipolar Perceived Caring Scale (Teven and McCroskey, 1997). The abbreviation was adopted to reduce respondent burden in a questionnaire containing the 36-item PRBT field pool and multiple intermediate-variable and outcome measures. Item selection was content-based rather than data-driven: one item represented empathy, two represented understanding, and two represented responsiveness. The five items were translated and cognitively tested using the procedure above and were answered on 5-point bipolar response formats. A one-factor CFA fit the Study 2 data adequately, χ^2^(5) = 10.84, comparative fit index (CFI) = 0.993, TLI = 0.986, RMSEA = 0.048, and standardized root mean square residual (SRMR) = 0.019. Nevertheless, adequate internal performance does not establish that five items exhaust the broader domain represented by the original 10-item instrument or by teacher caring more generally. Accordingly, this measure is treated throughout as an abbreviated comparison benchmark, and the discriminant- and incremental-validity findings are interpreted specifically relative to this operationalization. The administered item pairs and selection rationale are provided in Supplementary Table S3.

#### 3.2.3 Teacher–student relationship quality

Relationship quality was assessed with five student-report indicators adapted from the student version of the Teacher–Student Relationship Inventory (Ang et al., 2020), a development of the original inventory (Ang, 2005). The indicators sampled satisfaction, instrumental help, trust, and low conflict. Higher scores indicate a more positive relationship.

#### 3.2.4 Basic psychological need satisfaction

Five educational-context indicators were selected from the satisfaction items of the Basic Psychological Need Satisfaction and Frustration Scale (Chen et al., 2015), covering autonomy, competence, and relatedness. The short form was specified before data collection, and higher scores indicate greater aggregate need satisfaction.

#### 3.2.5 Learning motivation

Five indicators adapted from the intrinsic and identified-regulation portions of the Academic Motivation Scale (Vallerand et al., 1992) assessed willingness to invest effort for interest and personally endorsed value. The present study did not treat the score as the complete multidimensional AMS profile.

#### 3.2.6 School belonging

Five positively keyed indicators adapted from the Psychological Sense of School Membership scale (Goodenow, 1993) assessed acceptance, inclusion, and identification with the institution.

#### 3.2.7 Academic engagement

Five indicators adapted from the student version of the Utrecht Work Engagement Scale (Schaufeli et al., 2002) assessed vigor, dedication, and absorption. Academic engagement was analyzed as a secondary criterion and was not included in H1–H6.

#### 3.2.8 Marker variable and covariates

A four-item marker assessed neutral preferences for visual arrangement and routine that were not theoretically related to teacher behavior or student motivation. Gender, grade level, family socioeconomic status, and self-reported prior academic achievement were included as covariates. Exact item sources, scoring rules, and the marker wording are summarized in Supplementary Table S3.

### 3.3 Data analysis

Analyses were conducted in IBM SPSS Statistics 29 (IBM Corp., Armonk, NY, USA) and R 4.4.1 (R Foundation for Statistical Computing, Vienna, Austria) using psych 2.4.6, lavaan 0.6-19, and semTools 0.5-6. The bootstrap random seed was fixed at 20,260,318. Descriptive analyses used available item responses; latent-variable analyses used FIML under a missing-at-random assumption.

In Study 1, exploratory factor analysis used maximum-likelihood extraction and oblique promax rotation. Item screening and final factor retention were conducted sequentially. The initial 36-item pool was first evaluated for factorability and screened using primary loadings, cross-loadings, communalities, primary-to-secondary loading separation, source fidelity, and content coverage. Items were normally retained when the primary loading was at least 0.50, communality at least 0.40, the largest cross-loading below 0.30, and the primary-to-secondary loading difference at least 0.20. Borderline items could be retained only when source fidelity and content coverage would otherwise be weakened. After 12 items were removed, the exploratory factor analysis was re-estimated on the retained 24-item set. Factor retention for this final solution combined parallel analysis, the scree pattern, and theoretical interpretability. The complete bilingual wording and retention decisions for all 36 candidate items are reported in Supplementary Table S1; the full Study 1 cross-loading matrix and communalities, together with the Study 2 standardized CFA loadings for the retained 24 items, are reported in Supplementary Table S2.

In Study 2, confirmatory factor analyses compared a correlated six-factor PRBT model, a second-order model, a bifactor diagnostic model, and a one-factor model. Model fit was assessed with χ^2^, χ^2^/*df*, CFI, TLI, RMSEA, and SRMR (Hu and Bentler, 1999). The second-order model evaluated whether a broad PRBT composite was psychometrically defensible. The bifactor model was treated diagnostically rather than selected from global fit alone; omega hierarchical and the stability of specific factors were also considered. Convergent validity was assessed with standardized loadings, composite reliability (CR), and average variance extracted (AVE). Reliability was evaluated with Cronbach's α, McDonald's total ω, and, for the general score, hierarchical ω.

Discriminant validity (H5) was evaluated using the Fornell–Larcker criterion (Fornell and Larcker, 1981) and heterotrait–monotrait ratios (HTMT <0.85) (Henseler et al., 2015). Measurement invariance across gender and grade was tested in the configural → metric → scalar sequence, retaining invariance when ΔCFI ≤ 0.01 and ΔRMSEA ≤ 0.015 (Cheung and Rensvold, 2002). Grade invariance compared first/second-year students with third/fourth-year students to preserve adequate group sizes.

The focal structural analysis compared direct-association, single-intermediate-variable, parallel, theoretically ordered serial, and reverse-ordered serial specifications. AIC and BIC were emphasized for non-nested ordering comparisons, but no fit comparison was interpreted as establishing temporal precedence. Cross-sectional indirect associations were estimated with 5,000 case-level bias-corrected bootstrap resamples (Preacher and Hayes, 2008). H6 was tested with hierarchical regression entering covariates first, the abbreviated care-oriented teaching benchmark second, and PRBT third; Δ*R*^2^ and Cohen's *f*^2^ are reported. The same sequence was used for the exploratory academic-engagement criterion.

Common-method analyses were treated as sensitivity diagnostics, not as tests capable of ruling out bias. They included a marker-variable model, an unmeasured latent method construct (ULMC), and the Harman single-factor descriptive check (Podsakoff et al., 2003). Robustness checks used robust maximum likelihood, exclusion of flagged careless responders, alternative intermediate-variable specifications, and comparison of parallel and reverse chains. Analysis inputs and item-level results are provided in Supplementary material.

## 4 Results

### 4.1 Study 1: scale development and exploratory factor structure

The initial 36-item pool was factorable. The KMO measure was 0.93, and Bartlett's test of sphericity was significant, χ^2^(630) = 5128.74, *p* <0.001. The initial item-screening EFA, considered together with source fidelity and content coverage, identified 12 items for removal: six had a primary loading below 0.50, five showed a problematic cross-loading or a primary-to-secondary loading difference below 0.20, and one had redundant wording with weaker source fidelity. The resulting 24-item set contained four items per dimension.

The maximum-likelihood promax EFA was then re-estimated on the retained 24-item set. Parallel analysis of this final solution supported six factors: the sixth observed eigenvalue (2.01) exceeded the corresponding 95th-percentile random eigenvalue (0.97), whereas the seventh observed eigenvalue (0.78) was below its random counterpart (0.91). The first six eigenvalues were 4.28, 3.72, 3.34, 2.96, 2.42, and 2.01. Primary loadings ranged from 0.66 to 0.86, the largest cross-loadings ranged from 0.08 to 0.29, and communalities ranged from 0.48 to 0.73. The six-factor solution accounted for 78.01% of the variance in the retained item set. Dimension-level α ranged from 0.82 to 0.89 and ω from 0.83 to 0.90. Table 3 summarizes the retained 24-item solution, and Figure 2 presents its eigenvalue pattern. Supplementary Table S1 reports the bilingual wording and retention decisions for all 36 fielded items; Supplementary Table S2 reports the full Study 1 cross-loading matrix and communalities, together with the Study 2 standardized CFA loadings for the retained 24 items.

**Table 3:** Study 1 exploratory factor analysis summary for the retained 24-item PRBT scale (*N* = 326).

Factor	Items	Loading range	Eigenvalue	Variance %	Cumulative %	α	ω
F1 Benevolent concern	4	0.75–0.84	4.28	17.82	17.82	0.88	0.89
F2 Inclusive teaching	4	0.69–0.85	3.72	15.49	33.31	0.86	0.87
F3 Patient guidance	4	0.79–0.86	3.34	13.91	47.22	0.89	0.90
F4 Responsive instruction	4	0.73–0.78	2.96	12.34	59.56	0.85	0.86
F5 Norm-guided care	4	0.66–0.74	2.42	10.08	69.64	0.82	0.83
F6 Moral modeling	4	0.72–0.83	2.01	8.37	78.01	0.87	0.88

Extraction: maximum likelihood with promax rotation. Factorability statistics for the initial 36-item pool were KMO = 0.93 and Bartlett's χ^2^(630) = 5128.74, *p* <0.001. The eigenvalues and variance percentages shown in the table are from the re-estimated retained 24-item solution. Item codes are BC1–BC4, IT1–IT4, PG1–PG4, RI1–RI4, NGC1–NGC4, and MM1–MM4. Supplementary Table S1 reports the bilingual wording and retention decisions for all 36 fielded items; Supplementary Table S2 reports the full Study 1 cross-loading matrix and communalities, together with the Study 2 standardized CFA loadings for the retained 24 items.

**Figure 2:**
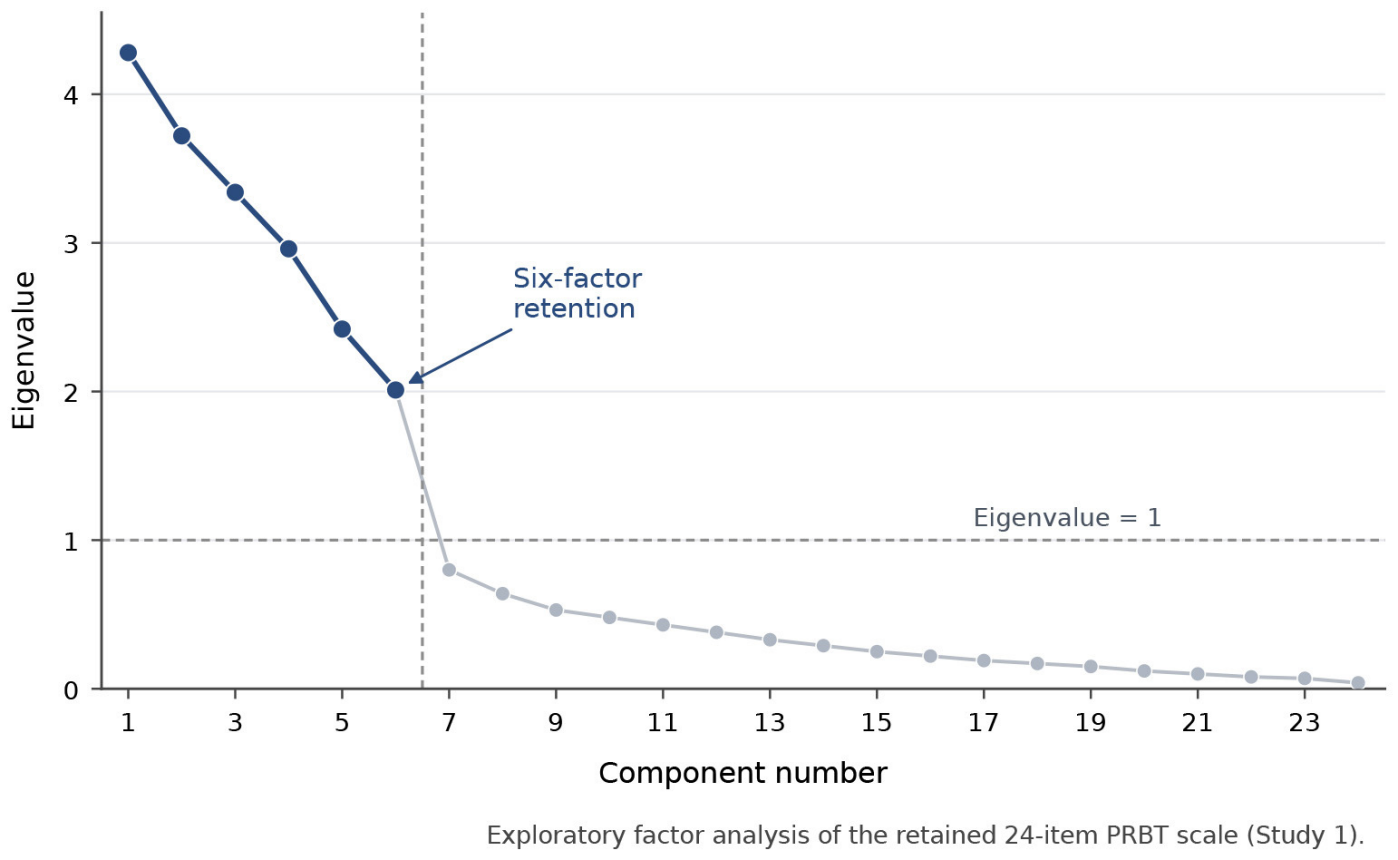
Scree plot of eigenvalues for the retained 24-item PRBT solution. The drop after the sixth eigenvalue is consistent with the six-factor retention supported by parallel analysis in Study 1.

### 4.2 Study 2: confirmatory factor analysis and measurement quality

The correlated six-factor PRBT model fit well, χ^2^(237) = 371.82, χ^2^/*df* = 1.57, CFI = 0.972, TLI = 0.966, RMSEA = 0.033, and SRMR = 0.035. A second-order model in which the six dimensions loaded on a general PRBT factor also fit adequately, although less well than the correlated-factor model, χ^2^(246) = 421.17, CFI = 0.963, TLI = 0.957, RMSEA = 0.039, and SRMR = 0.043. Second-order loadings ranged from 0.72 to 0.87. A bifactor diagnostic model showed marginally stronger global fit, χ^2^(228) = 348.92, CFI = 0.976, TLI = 0.969, RMSEA = 0.032, and SRMR = 0.031, but two specific factors had weak residual reliability after the general factor was controlled. Thus, no single representation was superior on every criterion: the bifactor model had the strongest global fit, the correlated six-factor model retained the clearest and most stable dimension interpretation, and the second-order model offered a defensible but somewhat less precise broad composite.

The full measurement model, including PRBT, care-oriented teaching, teacher–student relationship quality, basic psychological need satisfaction, learning motivation, school belonging, and academic engagement, fit well, χ^2^(833) = 1548.39, χ^2^/*df* = 1.86, CFI = 0.957, TLI = 0.949, RMSEA = 0.041, and SRMR = 0.039. A one-factor model fit poorly, and a diagnostic model combining PRBT with care-oriented teaching also fit less well, supporting treatment of the constructs as related but distinct. Table 4 reports the model comparisons.

**Table 4:** Study 2 measurement model fit indices (*N* = 512).

Model	χ^2^	df	χ^2^/*df*	CFI	TLI	RMSEA	SRMR
Correlated six-factor PRBT	371.82	237	1.57	0.972	0.966	0.033	0.035
Second-order PRBT	421.17	246	1.71	0.963	0.957	0.039	0.043
Bifactor diagnostic model	348.92	228	1.53	0.976	0.969	0.032	0.031
Full measurement model	1548.39	833	1.86	0.957	0.949	0.041	0.039
One-factor model	6124.65	902	6.79	0.681	0.657	0.107	0.119
PRBT and COT combined	2214.83	849	2.61	0.918	0.906	0.056	0.071

Reliability and convergent validity were satisfactory (Table 5). At the overall scale level, PRBT showed α = 0.93 and total ω = 0.94. Hierarchical ω = 0.81 indicated that a broad general score contained substantial reliable variance, while explained common variance was 0.64. The six second-order loadings and the correlated-factor solution nevertheless showed meaningful multidimensionality. We therefore recommend the total composite for research questions about overall perceived ren-based teaching and the six subscale means when the aim is dimension-specific diagnosis or intervention. Total and subscale scores should not be treated as interchangeable. Across dimensions, CR ranged from 0.80 to 0.88 and AVE from 0.50 to 0.65. The comparison construct, intermediate variables, and outcomes showed acceptable reliability and convergent validity.

**Table 5:** Reliability and convergent validity in Study 2 (*N* = 512).

Construct/ dimension	Items	CR	AVE	α	ω
PRBT total composite	24	–	–	0.93	0.94
Benevolent concern	4	0.85	0.60	0.84	0.85
Inclusive teaching	4	0.88	0.65	0.87	0.88
Patient guidance	4	0.87	0.63	0.86	0.87
Responsive instruction	4	0.83	0.56	0.82	0.83
Norm-guided care	4	0.85	0.6	0.84	0.85
Moral modeling	4	0.80	0.50	0.80	0.81
Care-oriented teaching	5	0.90	0.64	0.89	0.90
Teacher-student relationship quality	5	0.88	0.58	0.87	0.88
Basic psychological need satisfaction	5	0.87	0.57	0.86	0.87
Learning motivation	5	0.88	0.59	0.87	0.88
School belonging	5	0.89	0.61	0.88	0.89
Academic engagement	5	0.90	0.64	0.89	0.90

CR, composite reliability; AVE, average variance extracted. For the total composite, hierarchical ω = 0.81 and explained common variance = 0.64. Study 2 item-level standardized CFA loadings are reported in Supplementary Table S2.

### 4.3 Descriptive statistics, correlations, and discriminant validity (H5)

Figure 3 summarizes the construct-level descriptive profile and correlation structure. PRBT was positively related to all focal constructs, including teacher–student relationship quality (*r* = 0.67), basic psychological need satisfaction (*r* = 0.55), learning motivation (*r* = 0.51), school belonging (*r* = 0.56), and academic engagement (*r* = 0.48). Consistent with H5, PRBT correlated substantially with care-oriented teaching (*r* = 0.64), showing meaningful conceptual overlap. However, the discriminant-validity diagnostics supported distinctiveness. The HTMT ratio for PRBT and care-oriented teaching was 0.69, below the 0.85 criterion, and the largest HTMT value in the matrix was 0.76, also below 0.85. In the Fornell–Larcker check, the square roots of AVE exceeded the corresponding inter-construct correlations. H5 was therefore supported at the level of the measures used here: PRBT and the abbreviated care-oriented teaching benchmark were strongly related but empirically discriminable.

**Figure 3:**
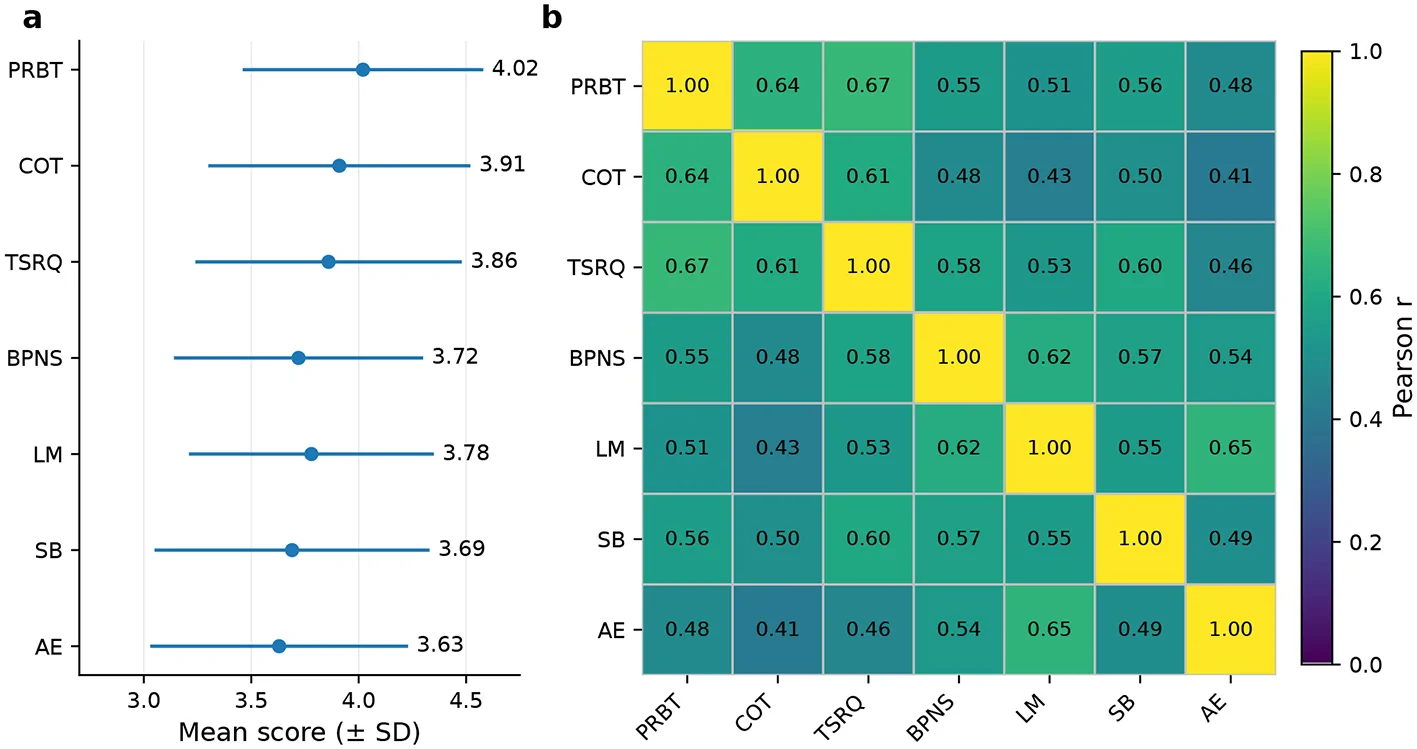
Construct-level descriptive profile and correlation structure in Study 2. **(a)** Shows mean scores with standard deviations for the focal constructs. **(b)** Shows Pearson correlations among PRBT, care-oriented teaching, intermediate variables, and student outcomes. PRBT, Perceived Confucian Ren-Based Teaching; COT, abbreviated Care-Oriented Teaching benchmark; TSRQ, Teacher–Student Relationship Quality; BPNS, Basic Psychological Need Satisfaction; LM, Learning Motivation; SB, School Belonging; AE, Academic Engagement.

### 4.4 Measurement invariance and multigroup structural stability

Measurement invariance was examined across gender and grade (Table 6). For gender, configural, metric, and scalar models met the fit-change criteria, with ΔCFI values from –0.002 to –0.003 and ΔRMSEA values within 0.002. Residual invariance showed partial support, which is not required for comparing latent structural relations. For grade, first/second-year students were compared with third/fourth-year students; configural, metric, and scalar invariance were also supported. Multigroup structural checks further showed that constraining all structural paths to equality did not materially reduce model fit for either gender (ΔCFI=-0.001) or grade (ΔCFI=-0.001), indicating that the focal structural relations were broadly comparable across groups.

**Table 6:** Measurement invariance across gender and grade (*N* = 512).

Grouping	Model	χ^2^	df	CFI	TLI	RMSEA	SRMR	ΔCFI	ΔRMSEA	Decision
Gender	Configural	1134.52	660	0.953	0.945	0.04	0.044			Acceptable
Gender	Metric	1161.84	678	0.951	0.945	0.039	0.046	–0.002	–0.001	Supported
Gender	Scalar	1198.22	696	0.948	0.943	0.041	0.047	–0.003	0.002	Supported
Gender	Residual	1247.63	720	0.944	0.941	0.042	0.049	–0.004	0.001	Partial support
Grade	Configural	1298.77	992	0.95	0.942	0.039	0.046			Acceptable
Grade	Metric	1337.18	1,016	0.948	0.942	0.039	0.047	–0.002	0	Supported
Grade	Scalar	1385.4	1,040	0.945	0.94	0.04	0.049	–0.003	0.001	Supported

### 4.5 Structural model and cross-sectional indirect associations (H1–H4)

Competing structural models did not identify one model as best on every fit statistic (Table 7). The parallel specification had marginally higher CFI and lower SRMR than the theoretically ordered serial model, whereas the serial model had the lowest AIC and BIC and matched the hypothesized distal-to-proximal ordering. The reverse-ordered model was less favorable on both information criteria. We therefore retained the serial specification as the focal theoretically ordered representation for estimating indirect associations. This choice concerns comparative statistical representation of same-occasion covariance; it does not establish that relationship quality temporally precedes need satisfaction or that either variable causally transmits an effect of PRBT.

**Table 7:** Comparison of alternative structural models in Study 2 (*N* = 512).

Model	χ^2^	df	χ^2^/*df*	CFI	TLI	RMSEA	SRMR	AIC	BIC
Direct associations only	1811.24	868	2.09	0.921	0.914	.051	0.061	58,126	58,402
TSRQ single intermediate	1738.40	865	2.01	0.943	0.937	0.045	0.050	57,910	58,203
BPNS single intermediate	1722.91	865	1.99	0.946	0.940	0.044	0.049	57,863	58,156
Parallel intermediates	1664.83	861	1.93	0.954	0.947	0.043	0.044	57,714	58,033
Theoretically ordered serial model	1677.31	862	1.95	0.952	0.945	0.043	0.046	**57,702**	**58,016**
Reverse-ordered serial model	1694.25	862	1.97	0.950	0.943	0.044	0.047	57,829	58,143

TSRQ, teacher–student relationship quality; BPNS, basic psychological need satisfaction. Bold values indicate the lowest AIC and BIC among the compared structural models.

The standardized paths are shown in Table 8 and Figure 4. PRBT was strongly associated with teacher–student relationship quality (β = 0.56, *p* <0.001). Teacher–student relationship quality was associated with need satisfaction (β = 0.48, *p* <0.001), learning motivation (β = 0.18, *p* = 0.001), and school belonging (β = 0.22, *p* <0.001). Need satisfaction was a strong proximal correlate of learning motivation (β = 0.41, *p* <0.001) and school belonging (β = 0.37, *p* <0.001). After accounting for these intermediate variables and the abbreviated care-oriented teaching benchmark, the direct paths from PRBT to learning motivation (β = 0.16, *p* = 0.004) and school belonging (β = 0.14, *p* = 0.007) remained small but significant, supporting H1 and H2.

**Table 8:** Standardized SEM path coefficients for the focal theoretically ordered model (*N* = 512).

Path	β	SE	*z*	*p*	95% CI L	95% CI U
PRBT → TSRQ	0.56	0.041	13.66	<0.001	0.48	0.64
TSRQ → BPNS	0.48	0.043	11.16	<0.001	0.4	0.56
TSRQ → Learning motivation	0.18	0.052	3.46	0.001	0.08	0.28
TSRQ → School belonging	0.22	0.05	4.4	<0.001	0.12	0.32
BPNS → Learning motivation	0.41	0.049	8.37	<0.001	0.32	0.5
BPNS → School belonging	0.37	0.047	7.87	<0.001	0.28	0.46
PRBT → Learning motivation	0.16	0.055	2.91	0.004	0.05	0.27
PRBT → School belonging	0.14	0.052	2.69	0.007	0.04	0.24
COT → Learning motivation	0.12	0.051	2.35	0.019	0.02	0.22
COT → School belonging	0.13	0.05	2.6	0.010	0.03	0.23

Model fit: χ^2^/*df* = 1.95, CFI = 0.952, TLI = 0.945, RMSEA = 0.043, SRMR = 0.046.

**Figure 4:**
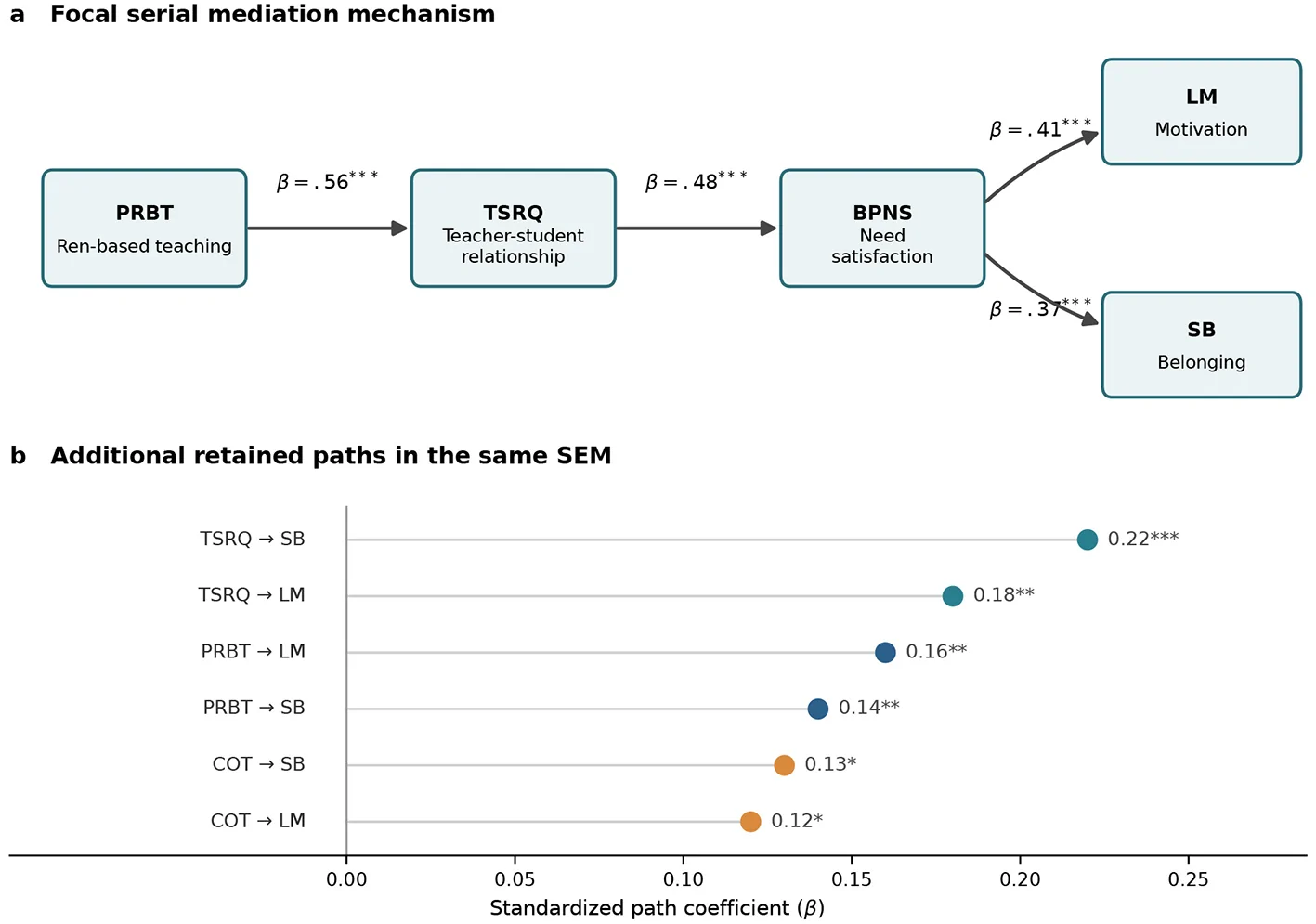
Empirical theoretically ordered indirect-association model and additional retained paths. **(a)** Shows the focal serial association pattern from PRBT to teacher–student relationship quality and basic psychological need satisfaction, which are in turn associated with learning motivation and school belonging. **(b)** Summarizes additional retained structural paths in the same SEM. Standardized coefficients are shown. PRBT, Perceived Confucian Ren-Based Teaching; TSRQ, Teacher–Student Relationship Quality; BPNS, Basic Psychological Need Satisfaction; LM, Learning Motivation; SB, School Belonging; COT, abbreviated Care-Oriented Teaching benchmark. The cross-sectional paths represent modeled associations, not observed temporal or causal transitions. ^*^p <0.05, ^**^p <0.01, ^***^p <0.001.

Bootstrap estimates of the pre-specified indirect associations were statistically consistent with H3 and H4 (Figure 5). The indirect association PRBT → teacher–student relationship quality → learning motivation was positive, estimate = 0.101, 95% CI [0.047, 0.169], and the corresponding indirect association with school belonging was also positive, estimate = 0.123, 95% CI [0.062, 0.192]. The serial indirect association PRBT → teacher–student relationship quality → need satisfaction → learning motivation was positive, estimate = 0.110, 95% CI [0.063, 0.168], as was the corresponding serial association with school belonging, estimate = 0.099, 95% CI [0.054, 0.157]. The total modeled associations were 0.371 for learning motivation and 0.362 for school belonging. H3 and H4 were therefore supported only in the stated cross-sectional sense: the observed covariance pattern was compatible with the hypothesized ordering, but temporal mediation and causal transmission were not established.

**Figure 5:**
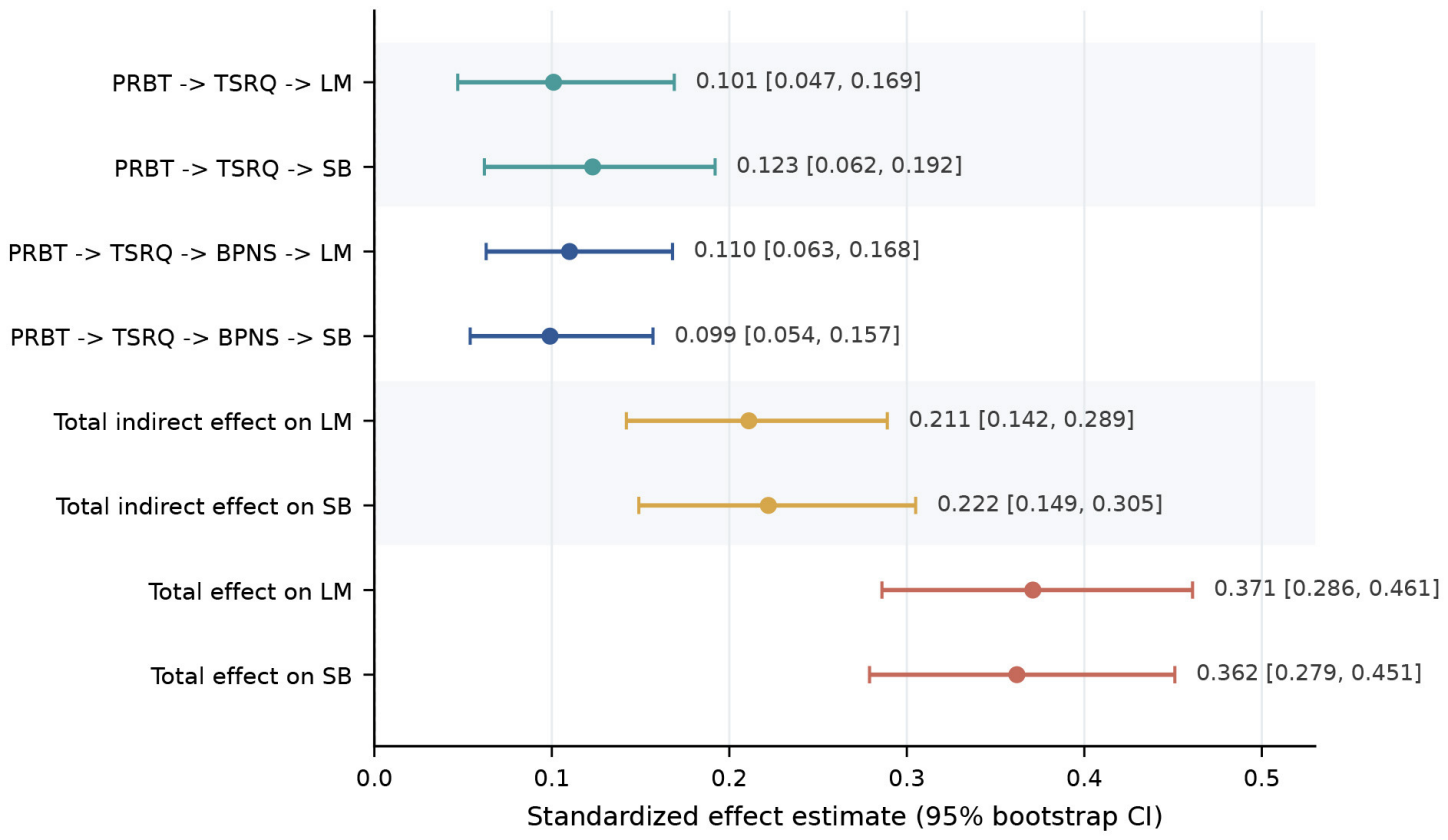
Bootstrap indirect and total associations in the theoretically ordered structural model. Points denote standardized estimates and horizontal intervals denote 95% bias-corrected bootstrap confidence intervals based on 5,000 resamples. Intervals that do not cross zero indicate statistically reliable modeled associations; because all variables were measured on one occasion, they should not be interpreted as evidence of temporal mediation.

### 4.6 Incremental association relative to the abbreviated care-oriented teaching benchmark (H6)

Hierarchical regressions tested whether PRBT accounted for additional variance after demographic covariates and the abbreviated care-oriented teaching benchmark were entered (Figure 6). For learning motivation, the covariates explained *R*^2^ = 0.118. Adding the care-oriented benchmark increased *R*^2^ to 0.318, and adding PRBT further increased *R*^2^ to 0.379, Δ*R*^2^ = 0.061, *f*^2^ = 0.098. In the final model, PRBT retained a significant association with learning motivation (β = 0.31, *p* <0.001), as did the care-oriented benchmark (β = 0.20, *p* <0.001). For school belonging, covariates explained *R*^2^ = 0.104; the care-oriented benchmark increased *R*^2^ to 0.354; and PRBT further increased *R*^2^ to 0.423, Δ*R*^2^ = 0.069, *f*^2^ = 0.120. PRBT again retained a unique association (β = 0.34, *p* <0.001). The separate academic-engagement analysis addressed the exploratory question. Covariates explained *R*^2^ = 0.089, the care-oriented benchmark increased *R*^2^ to 0.265, and PRBT increased it to 0.318, Δ*R*^2^ = 0.053, *f*^2^ = 0.078. In the final model, PRBT remained associated with engagement (β = 0.29, *p* <0.001). H6 was supported for the two focal outcomes relative to the abbreviated benchmark, and the engagement result provided secondary evidence of nomological breadth. These regressions do not establish that PRBT would show the same increment over the complete Perceived Caring Scale or over other comprehensive teacher-care measures.

**Figure 6:**
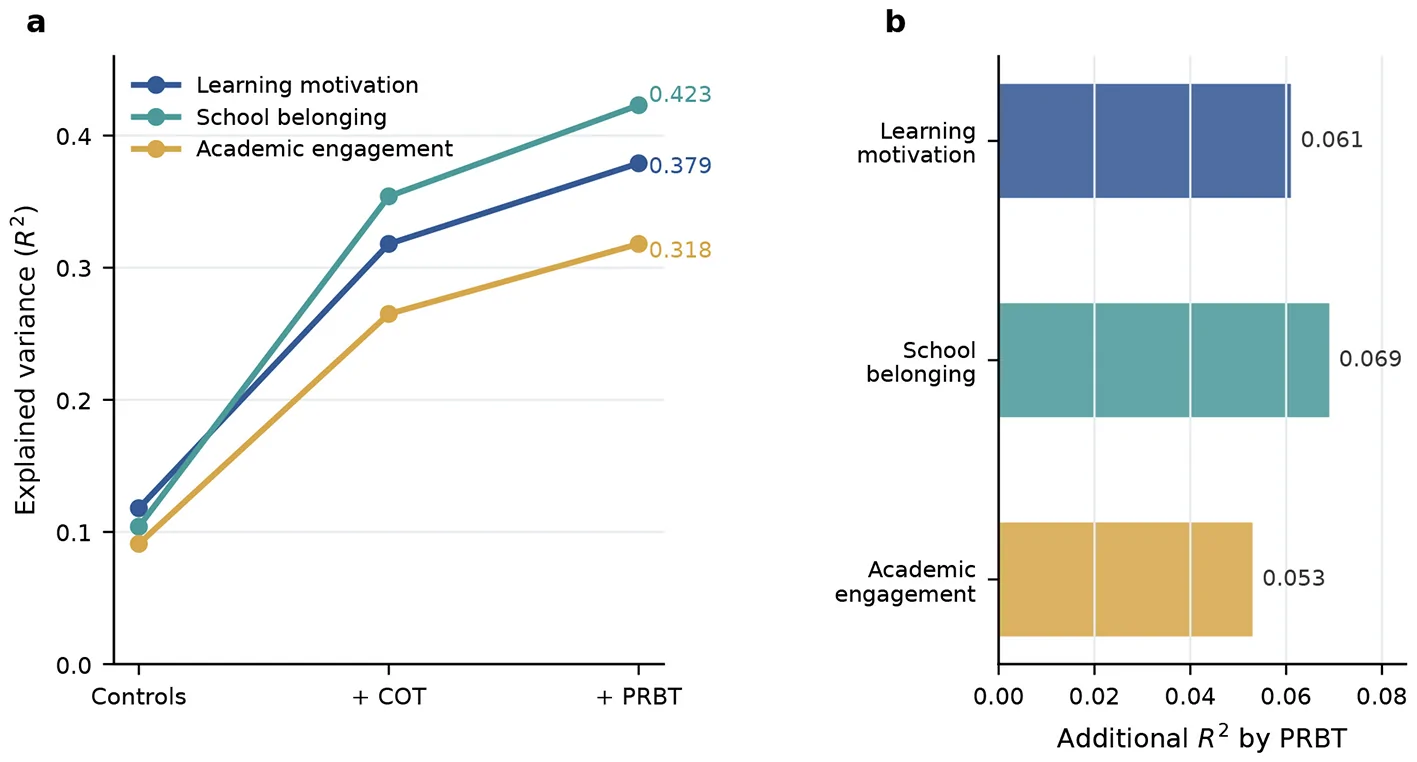
Incremental association of PRBT relative to demographic covariates and the abbreviated care-oriented teaching benchmark. **(a)** Shows the change in explained variance across the three hierarchical regression steps. **(b)** Summarizes the additional variance associated with PRBT after covariates and the abbreviated benchmark were entered.

### 4.7 Common-method variance, robustness, and sensitivity

Harman's first unrotated factor accounted for 27.4% of total item variance, the ULMC model attributed approximately 0.18 of indicator variance to a common method factor, and marker-variable correlations with focal constructs ranged from −0.06 to 0.08. Adding the ULMC changed focal standardized paths by no more than 0.02. These diagnostics indicate that the observed covariance structure was not reducible to one dominant estimated response factor, but they cannot rule out shared-method inflation. In particular, a generally favorable perception of the focal teacher, transient affect, consistency motives, or other same-source processes could elevate PRBT, relationship quality, need satisfaction, and outcome ratings together. This possibility is especially consequential for the indirect associations because bootstrap confidence intervals address sampling uncertainty, not bias created by same-source, same-occasion covariance. The reported indirect-association magnitudes should therefore be treated as method-contingent cross-sectional estimates rather than method-free evidence of mediation.

Robustness estimates were stable in magnitude (Table 9): the PRBT → teacher–student relationship coefficient was 0.56 in the primary SEM, 0.54 with the marker variable, 0.55 with robust maximum likelihood, and 0.57 after exclusion of flagged careless responders. The parallel model slightly improved CFI and SRMR, whereas the theoretically ordered serial specification retained lower AIC and BIC. The model evidence is therefore consistent with, but not uniquely diagnostic of, the proposed ordering.

**Table 9:** Robustness and common-method checks.

Check	Target estimate	Estimate	SE	95% CI L	95% CI U	Interpretation
Primary SEM	β PRBT → TSRQ	0.56	0.041	0.48	0.64	Stable
With marker variable	β PRBT → TSRQ	0.54	0.043	0.46	0.62	Stable
Robust ML estimator	β PRBT → TSRQ	0.55	0.042	0.47	0.63	Stable
Excluding careless responses	β PRBT → TSRQ	0.57	0.04	0.49	0.65	Stable
Controlling academic engagement	PRBT total association with LM	0.35	0.047	0.26	0.44	Stable
Alternative outcome model	PRBT total association with SB	0.34	0.048	0.25	0.43	Stable
CMV ULMC comparison	Common factor variance	0.18				Descriptive sensitivity only
Harman single factor	First factor variance	0.27				Does not rule out method bias

## 5 Discussion

This research developed and validated a student-rated measure of Confucian ren-based teaching, with explicit distinctions between education-specific textual anchors, pedagogical applications of broader Confucian principles, and interpretive synthesis from the *Analects*. Three findings stand out. First, PRBT formed a coherent, reliably measured, six-dimensional construct and was positively associated with students' learning motivation and school belonging. Second, the observed covariance pattern was consistent with a theoretically ordered relational–motivational account: higher PRBT was associated with higher teacher–student relationship quality, which was associated with greater basic psychological need satisfaction, which was in turn associated with motivation and belonging. The ordered serial model had the most favorable information criteria, but a parallel specification performed marginally better on some global fit indices; with same-occasion data, neither result establishes temporal mediation. Third, PRBT was strongly related to but empirically distinct from the five-item abbreviated care-oriented teaching benchmark and accounted for additional variance in the focal outcomes after that benchmark and demographic covariates were entered. This last finding is evidence of incremental association relative to the measure used here, not evidence that PRBT subsumes or outperforms the broader construct of teacher caring.

### 5.1 Theoretical contributions

The first contribution is a measurement contribution. PRBT does not derive its distinctiveness from the claim that benevolence, patience, or responsiveness are uniquely Confucian; those dimensions overlap substantively with established teacher-care and teacher-support constructs. Its added theoretical content is configurational. The scale places these relational behaviors alongside inclusive obligation, norm-guided benevolence, moral exemplarity, and a role-structured orientation toward students' broader cultivation. The source map further makes clear that the six dimensions do not all have the same textual status: inclusive teaching and patient guidance have education-specific textual anchors, benevolent concern, norm-guided care, and moral modeling apply broader Confucian principles pedagogically, and responsive instruction is an interpretive synthesis. This differentiated source claim strengthens construct transparency while avoiding the suggestion that contemporary psychological labels are literal categories in the classical text.

The measurement results provide initial evidence that this configuration is empirically coherent and distinguishable from the abbreviated care-oriented teaching benchmark used here. The strong PRBT–care correlation confirms substantial shared relational content, while the discriminant-validity tests and hierarchical regressions indicate that the two operationalizations are not statistically redundant in this sample. These findings do not show that teacher-care theory as a whole lacks the PRBT content or that PRBT would necessarily add the same variance beyond a full-length care measure. They instead establish a narrower claim: PRBT captures reliable variance not represented by the particular abbreviated benchmark administered in Study 2. The factor analyses also clarify score use. A general composite is defensible for broad research questions because the second-order loadings and hierarchical omega were adequate, but the correlated-factor solution remained clearer and more stable for dimension-level interpretation. Researchers should therefore use the total mean for overall PRBT and the six subscales for dimension-specific questions rather than treating them as interchangeable. This extends the philosophical comparison of *ren* and care (Li, 1994; Star, 2002; Tao, 2000) into empirical measurement.

The second contribution is to the cross-cultural study of teacher care. Within these Hubei post-secondary samples, a culturally grounded model of humane teaching was associated with the same focal outcomes as the abbreviated care-oriented benchmark while retaining statistically distinct variance. This pattern supports treating care-oriented and ren-based teaching as overlapping lenses rather than as either synonyms or mutually exclusive cultural alternatives. It also illustrates why cross-cultural measurement should examine whether a familiar instrument fully represents the locally theorized content domain before assuming conceptual equivalence. Broader claims about Chinese students as a whole, or about other Confucian-heritage contexts, require replication.

The third contribution concerns the fit between PRBT and an SDT-informed relational account. The cross-sectional indirect associations through relationship quality and need satisfaction are compatible with SDT's expectation that supportive relational contexts covary with need satisfaction and adaptive motivation (Ryan and Deci, 2000; Niemiec and Ryan, 2009; Chen et al., 2015). Need satisfaction was the strongest proximal correlate of both focal outcomes, while the remaining direct paths from PRBT were comparatively small. However, compatibility with the hypothesized covariance pattern is not a test of temporal mechanism: relationship quality and need satisfaction may be reciprocal, jointly determined, or partly consequences of students' broader positive appraisals. The secondary engagement result extends the nomological pattern to behaviorally expressed involvement, but it remains exploratory because engagement was not part of the focal hypothesis set.

### 5.2 Practical implications

For post-secondary teachers and teacher educators working in settings similar to the three Hubei institutions studied here, the dimensional structure offers culturally legible foci for reflection: caring for students as persons, teaching inclusively, guiding patiently, differentiating instruction, exercising warmth within fair expectations, and modeling the values one asks of students. Norm-guided care and moral modeling are especially useful for articulating aspects of the proposed Confucian configuration that are less explicit in measures centered primarily on empathy, understanding, and responsiveness. These implications remain provisional: the present evidence is associational, comes from convenience samples in one province, and should not be generalized to all Chinese or Confucian-heritage educational settings without replication and intervention-based testing.

### 5.3 Limitations and future directions

Several limitations bound the conclusions. First, the design is cross-sectional. The serial model had lower AIC and BIC than the reverse chain, but the parallel model showed marginally stronger CFI and SRMR, and none of these comparisons establishes temporal precedence. The indirect associations should therefore be interpreted as theoretically ordered cross-sectional associations, not as evidence that PRBT first changes relationship quality, which then changes need satisfaction. A four-wave design measuring PRBT, relationship quality, need satisfaction, and outcomes at separate occasions would provide a stronger test, as would intervention or quasi-experimental work.

Second, PRBT, care-oriented teaching, the intermediate variables, and the outcomes were reported by the same student in the same questionnaire. Harman, marker-variable, and ULMC analyses are limited diagnostics and cannot eliminate common-method bias. A global positive view of the teacher, transient affect, or consistency motives may inflate both direct and indirect associations. This concern is especially important for indirect-association estimates because bootstrap intervals quantify sampling uncertainty but do not correct shared-method covariance. Future studies should combine student ratings with teacher reports, independent classroom observation, attendance or learning-platform behavior, and administrative indicators, ideally across multiple waves.

Third, the samples were convenience samples from three post-secondary institution types in one Chinese province. Representation across comprehensive, normal, and vocational institutions is useful, but it does not make the samples regionally or nationally representative. Confucian educational practices and the meaning of benevolent authority may vary across institutional cultures, regions, school levels, and national contexts. Replication should use probability-based or stratified multi-province samples and test invariance across secondary, vocational, and higher-education settings as well as other Confucian-heritage and non-Confucian contexts.

Fourth, the five-item care-oriented teaching measure was an abbreviated form of the original 10-item bipolar instrument. It showed good internal and factorial performance here, but satisfactory one-factor structure and reliability in the present Chinese sample do not establish cross-cultural measurement equivalence with the original North American instrument. The abbreviated measure may also underrepresent the comparison construct and could make PRBT's incremental contribution appear larger than it would against a fuller care measure. Replication should compare PRBT with the complete Perceived Caring Scale and with additional constructs such as autonomy-supportive teaching, teacher immediacy, and instructional support.

Fifth, the PRBT scale requires further psychometric scrutiny. Test–retest reliability, longitudinal predictive validity, sensitivity to intervention, and measurement invariance across regions and cultures remain unknown. The higher-order results support qualified use of an overall score, but the correlated six-factor model retained the clearest dimension structure; total and subscale scores should therefore be selected according to the research question.

Finally, the six PRBT dimensions differ in their proximity to the classical text. Inclusive teaching and patient guidance have education-specific textual anchors; benevolent concern, norm-guided care, and moral modeling are contemporary pedagogical applications of broader Confucian principles; and responsive instruction is an interpretive synthesis of differentiated guidance in *Analects* 11.22 and 6.21. The later expression *yin cai shi jiao* should not be presented as verbatim canonical wording. These source-status distinctions are part of the construct definition rather than merely a translation issue. Future content-validity studies should include broader panels of classical-text scholars and teachers and examine whether the proposed interpretations and dimensions are understood similarly across regions and Confucian-heritage settings.

## 6 Conclusion

Across two convenience samples of post-secondary students from three institutions in Hubei Province, this study developed and validated a six-dimensional Perceived Confucian Ren-Based Teaching scale and provided initial evidence for its nomological value. PRBT was strongly related to but empirically distinguishable from the abbreviated care-oriented teaching benchmark used in Study 2, and it accounted for additional variance in learning motivation and school belonging after that benchmark and demographic covariates were entered. Its associations with the focal outcomes were also statistically compatible with a theoretically ordered indirect pattern involving teacher–student relationship quality and basic psychological need satisfaction. Because all focal variables were measured by the same students on one occasion, this pattern should be interpreted as cross-sectional indirect association rather than causal mediation. PRBT is therefore best understood at this stage as a culturally grounded, overlapping, and potentially complementary operationalization of humane teaching whose distinctiveness is configurational rather than based on uniquely Confucian ownership of caring behaviors. Replication with full-length comparison measures, multi-wave and multi-source designs, and broader regional and cultural samples is needed before stronger causal, incremental, or general claims are warranted.

## Data Availability

The raw data supporting the conclusions of this article will be made available by the authors, without undue reservation.
