# Supplementary material for: Perceived Confucian Ren-Based Teaching: scale development, construct validation, and associations with learning motivation and school belonging
Source: Front Psychol. 2026 Sep 30;17:1898379. doi: 10.3389/fpsyg.2026.1898379 (PMC13661964; doi:10.3389/fpsyg.2026.1898379)
Supplement: Supplementary file 1 [file Data_Sheet_1.zip › Supplementary_Materials/PRBT_Supplementary_Materials/PRBT_Supplementary_Material.pdf]

Supplementary Materials

Perceived Confucian *Ren*-Based Teaching: Scale Development, Construct Validation, and Associations With Learning Motivation and School Belonging

Wei Lu
College of Literature, Hubei University of Education, Wuhan, Hubei, China

Study context. Study 1 included 326 postsecondary students and was conducted from October to November 2025. Study 2 included 512 postsecondary students and was conducted from March to April 2026. Participants were recruited from Jianghan University, Hubei University of Education, and Hubei Urban Construction Vocational and Technical College in Hubei Province, China. The Institutional Ethics Committee of Hubei University of Education approved both studies (HUE-25163).

Analysis. Analyses used IBM SPSS Statistics 29 and R 4.4.1 with `psych` 2.4.6, `lavaan` 0.6-19, and `semTools` 0.5-6. Indirect effects used 5,000 case-level bias-corrected bootstrap resamples with seed 20260318. Latent-variable analyses used full-information maximum likelihood.

Supplementary Table S1. Complete 36-item PRBT development pool

Part A. Retained 24-item scale.

Code	Dimension	*Analects* basis	English wording	Original Chinese wording	Decision
BC1	Benevolent concern	Analects 12.22: ren as loving others	My teacher cares about students' overall growth, not only grades.	我的老师关心学生的全面成长，而不仅仅是成绩。	Retained
BC2	Benevolent concern	Analects 12.22: ren as loving others	My teacher pays attention to students' well-being as well as their academic work.	我的老师既关注学生的学习，也关注学生的身心状态。	Retained
BC3	Benevolent concern	Analects 12.22: ren as loving others	When students encounter difficulties, my teacher offers sincere concern and help.	当学生遇到困难时，我的老师会真诚地给予关心和帮助。	Retained
BC4	Benevolent concern	Analects 12.22: ren as loving others	My teacher treats each student as a person with distinctive needs and value.	我的老师把每位学生都当作具有独特需要和价值的人来对待。	Retained
IT1	Inclusive teaching	Analects 15.39: teaching without class distinctions	My teacher gives equal learning opportunities to students with different backgrounds or abilities.	我的老师为不同背景或能力的学生提供平等的学习机会。	Retained
IT2	Inclusive teaching	Analects 15.39: teaching without class distinctions	My teacher does not treat students differently because of achievement, family background, or status.	我的老师不会因为学生的成绩、家庭背景或身份而区别对待。	Retained
IT3	Inclusive teaching	Analects 15.39: teaching without class distinctions	My teacher encourages every student to participate in classroom learning.	我的老师鼓励每一位学生参与课堂学习。	Retained
IT4	Inclusive teaching	Analects 15.39: teaching without class distinctions	My teacher respects different viewpoints and ways of learning.	我的老师尊重学生不同的观点和学习方式。	Retained
PG1	Patient guidance	Analects 7.2: teaching without weariness	My teacher explains patiently when students have difficulty understanding.	当学生难以理解时，我的老师会耐心讲解。	Retained
PG2	Patient guidance	Analects 7.2: teaching without weariness	My teacher continues to provide guidance even when a student progresses slowly.	即使学生进步较慢，我的老师也会继续给予指导。	Retained
PG3	Patient guidance	Analects 7.2: teaching without weariness	When students make mistakes, my teacher patiently helps them improve.	当学生犯错时，我的老师会耐心帮助他们改进。	Retained
PG4	Patient guidance	Analects 7.2: teaching without weariness	My teacher is willing to explain important points repeatedly until students understand them.	我的老师愿意反复解释重要内容，直到学生理解。	Retained
RI1	Responsive instruction	Interpretive synthesis of Analects 11.22 and 6.21	My teacher adjusts guidance according to different students' learning needs.	我的老师会根据不同学生的学习需要调整指导方式。	Retained
RI2	Responsive instruction	Interpretive synthesis of Analects 11.22 and 6.21	My teacher uses different explanations according to students' levels of understanding.	我的老师会根据学生的理解程度采用不同的讲解方法。	Retained
RI3	Responsive instruction	Interpretive synthesis of Analects 11.22 and 6.21	My teacher offers targeted suggestions for the particular difficulties students encounter.	我的老师会针对学生遇到的具体困难提供相应建议。	Retained
RI4	Responsive instruction	Interpretive synthesis of Analects 11.22 and 6.21	My teacher adjusts learning tasks or support according to students' characteristics.	我的老师会根据学生的特点调整学习任务或支持方式。	Retained
NGC1	Norm-guided care	Analects 12.1: self-restraint and ritual propriety	My teacher's care has clear principles rather than simply indulging students.	我的老师的关心有明确原则，而不是一味迁就学生。	Retained
NGC2	Norm-guided care	Analects 12.1: self-restraint and ritual propriety	My teacher maintains reasonable classroom norms while showing concern for students.	我的老师在关心学生的同时坚持合理的课堂规范。	Retained
NGC3	Norm-guided care	Analects 12.1: self-restraint and ritual propriety	When a student violates a rule, my teacher responds in a respectful but firm manner.	当学生违反规则时，我的老师会以尊重但坚定的方式处理。	Retained
NGC4	Norm-guided care	Analects 12.1: self-restraint and ritual propriety	My teacher explains the reasons for classroom rules and helps students take responsibility.	我的老师会说明课堂规则背后的理由，并帮助学生承担责任。	Retained
MM1	Moral modeling	Analects 12.1, 13.6, and 14.42: cultivation and personal example	My teacher influences students through consistent moral example.	我的老师通过始终如一的道德行为影响学生。	Retained
MM2	Moral modeling	Analects 12.1, 13.6, and 14.42: cultivation and personal example	My teacher tries to practice the conduct that is expected of students.	我的老师要求学生做到的事情，自己也会努力做到。	Retained
MM3	Moral modeling	Analects 12.1, 13.6, and 14.42: cultivation and personal example	My teacher demonstrates honesty, fairness, and responsibility in words and actions.	我的老师在言行中表现出诚实、公正和责任感。	Retained
MM4	Moral modeling	Analects 12.1, 13.6, and 14.42: cultivation and personal example	My teacher provides students with an example through personal self-cultivation.	我的老师以自身的修养为学生树立榜样。	Retained

Part B. Items removed after Study 1 screening and item-specific decision basis.

Code	Dimension	English wording	Original Chinese wording	Decision	Decision basis
BC5	Benevolent concern	My teacher is concerned about every aspect of students' lives.	我的老师关心学生生活中的所有方面。	Removed after Study 1 screening	Primary loading below .50. Broad scope extended beyond the course-based teacher referent.

Continued on next page

Supplementary Table S1, Part B (continued)

**Code**	**Dimension**	**English wording**	**Original Chinese wording**	**Decision**	**Decision basis**
BC6	Benevolent concern	My teacher treats students as if they were members of the teacher’s family.	我的老师像对待家人一样对待学生。	Removed after Study 1 screening	Problematic cross-loading or insufficient loading separation. The family metaphor overlapped general warmth and moral-role perceptions.
IT5	Inclusive teaching	My teacher gives every student exactly the same treatment.	我的老师对每一位学生采取完全相同的对待方式。	Removed after Study 1 screening	Problematic cross-loading or insufficient loading separation. Identical treatment conflicted with learner-responsive differentiation.
IT6	Inclusive teaching	My teacher never gives special attention to high-achieving students.	我的老师从不对成绩优秀的学生给予特殊关注。	Removed after Study 1 screening	Primary loading below .50. Absolute and negatively framed wording reduced clarity.
PG5	Patient guidance	My teacher never becomes impatient with students.	我的老师对学生从不失去耐心。	Removed after Study 1 screening	Primary loading below .50. Absolute wording produced restricted response variation.
PG6	Patient guidance	My teacher repeats explanations whenever necessary without considering class progress.	无论课堂进度如何，我的老师都会在需要时反复讲解。	Removed after Study 1 screening	Problematic cross-loading or insufficient loading separation. The item combined patience with disregard for class progress.
RI5	Responsive instruction	My teacher changes teaching methods for every individual student.	我的老师会为每一位学生改变教学方法。	Removed after Study 1 screening	Primary loading below .50. The absolute individualization standard was unrealistic.
RI6	Responsive instruction	My teacher sets different learning requirements for students according to ability.	我的老师会根据学生能力设置不同的学习要求。	Removed after Study 1 screening	Problematic cross-loading or insufficient loading separation. The item could represent unequal expectations rather than responsive support.
NGC5	Norm-guided care	My teacher is strict because strictness itself is a form of care.	我的老师严格要求学生，因为严格本身就是一种关心。	Removed after Study 1 screening	Primary loading below .50. The wording could be interpreted as endorsing authoritarian strictness.
NGC6	Norm-guided care	My teacher relaxes rules when students are experiencing difficulties.	当学生遇到困难时，我的老师会放宽规则。	Removed after Study 1 screening	Problematic cross-loading or insufficient loading separation. The item mixed compassionate judgment with rule relaxation.
MM5	Moral modeling	My teacher is morally perfect both inside and outside the classroom.	我的老师在课堂内外都是道德上完美的人。	Removed after Study 1 screening	Primary loading below .50. Moral perfection was absolute and strongly halo-prone.
MM6	Moral modeling	My teacher’s private life provides a model for students.	我的老师的私人生活为学生提供了榜样。	Removed after Study 1 screening	Redundant wording with weaker source fidelity. The item extended beyond observable teaching behavior and added little coverage.

Note. Part A reports the 24 retained items and Part B reports the 12 non-retained items with item-specific reasons. Responsive instruction is an interpretive synthesis of *Analects* 11.22 and 6.21 rather than a verbatim classical formulation. The initial empirical screening removed six items with primary loadings below .50, five items with problematic cross-loadings or insufficient loading separation, and one redundant item with weaker source fidelity.

Supplementary Table S2. Retained-item EFA cross-loadings, communalities, and CFA loadings

Item	Dimension	F1 BC	F2 IT	F3 PG	F4 RI	F5 NGC	F6 MM	h^2	CFA λ	SE
BC1	Benevolent concern	0.80	0.04	0.21	0.16	0.21	0.03	0.73	0.75	0.03
BC2	Benevolent concern	0.79	0.11	0.04	0.18	0.10	0.25	0.69	0.78	0.04
BC3	Benevolent concern	0.84	0.22	0.16	0.21	0.04	0.23	0.73	0.70	0.04
BC4	Benevolent concern	0.75	0.06	0.11	0.02	0.19	0.09	0.61	0.85	0.03
IT1	Inclusive teaching	0.05	0.85	0.16	0.13	0.05	0.05	0.73	0.81	0.04
IT2	Inclusive teaching	0.21	0.81	0.09	0.07	0.03	0.10	0.73	0.85	0.04
IT3	Inclusive teaching	0.12	0.69	0.02	0.10	0.09	0.11	0.57	0.82	0.04
IT4	Inclusive teaching	0.13	0.72	0.18	0.15	0.19	0.04	0.60	0.75	0.03
PG1	Patient guidance	0.23	0.22	0.79	0.03	0.25	0.22	0.70	0.83	0.03
PG2	Patient guidance	0.25	0.20	0.80	0.06	0.13	0.20	0.68	0.81	0.03
PG3	Patient guidance	0.03	0.15	0.86	0.04	0.11	0.24	0.73	0.73	0.03
PG4	Patient guidance	0.19	0.03	0.82	0.20	0.13	0.23	0.72	0.80	0.04
RI1	Responsive instruction	0.23	0.19	0.14	0.78	0.07	0.09	0.67	0.69	0.03
RI2	Responsive instruction	0.21	0.06	0.10	0.73	0.06	0.11	0.63	0.77	0.04
RI3	Responsive instruction	0.06	0.22	0.18	0.74	0.07	0.14	0.61	0.70	0.03
RI4	Responsive instruction	0.16	0.04	0.20	0.78	0.23	0.06	0.66	0.81	0.03
NGC1	Norm-guided care	0.05	0.08	0.14	0.09	0.66	0.16	0.48	0.83	0.04
NGC2	Norm-guided care	0.16	0.09	0.22	0.08	0.74	0.09	0.60	0.73	0.04
NGC3	Norm-guided care	0.15	0.04	0.13	0.04	0.73	0.23	0.57	0.83	0.04
NGC4	Norm-guided care	0.11	0.08	0.17	0.10	0.71	0.29	0.58	0.69	0.03
MM1	Moral modeling	0.23	0.09	0.15	0.04	0.07	0.83	0.73	0.72	0.04
MM2	Moral modeling	0.03	0.03	0.04	0.15	0.10	0.72	0.57	0.72	0.04
MM3	Moral modeling	0.17	0.19	0.23	0.09	0.08	0.83	0.72	0.69	0.04
MM4	Moral modeling	0.12	0.21	0.02	0.20	0.21	0.73	0.59	0.71	0.04

Note. Study 1 used maximum-likelihood extraction with promax rotation. For the initial 36-item pool, KMO $=$ .93 and Bartlett's test was significant, $\chi^2(630) = 5128.74$, $p < .001$. In the retained 24-item solution, primary loadings ranged from .66 to .86, maximum cross-loadings from .08 to .29, and communalities from .48 to .73. Study 2 CFA loadings are standardized.

Supplementary Table S3. Measure sources, scoring, care-oriented teaching items, and marker wording

Construct	Source	Codes	Response	Scoring	Coverage and use
PRBT	Newly developed scale	BC1-BC4; IT1-IT4; PG1-PG4; RI1-RI4; NGC1-NGC4; MM1-MM4	1-5 agreement	Mean of four items for each dimension; total is the mean of six dimension scores when at least five are available	No retained item is reverse scored
Care-oriented teaching	Teven and McCroskey (1997), five-item abbreviated form	CAR1-CAR5	1-5 bipolar	Mean of five item pairs; higher values indicate greater perceived care	One empathy, two understanding, and two responsiveness indicators
Teacher-student relationship quality	Student Teacher-Student Relationship Inventory; Ang (2005); Ang, Ong, and Li (2020)	TSRQ1-TSRQ5	1-5 agreement	Mean score; higher values indicate a more positive relationship	Satisfaction, instrumental help, trust, and low conflict
Basic psychological need satisfaction	Basic Psychological Need Satisfaction and Frustration Scale; Chen et al. (2015)	BPNS1-BPNS5	1-5 agreement	Mean score	Autonomy, competence, and relatedness satisfaction
Learning motivation	Academic Motivation Scale; Vallerand et al. (1992)	LM1-LM5	1-5 agreement	Mean score	Intrinsic interest and identified value; not the full multidimensional AMS profile
School belonging	Psychological Sense of School Membership; Goodenow (1993)	SB1-SB5	1-5 agreement	Mean score	Acceptance, inclusion, and institutional identification
Academic engagement	Student Utrecht Work Engagement Scale; Schaufeli et al. (2002)	AE1-AE5	1-5 agreement	Mean score	Vigor, dedication, and absorption; secondary criterion
Marker variable	Neutral visual-arrangement and routine preferences	MK1-MK4	1-5 agreement	Mean score	Used only in common-method sensitivity analysis

Code	Domain	English wording or anchors	Chinese wording or anchors	Selection rationale
CAR1	Empathy	Does not care about me / Cares about me	不关心我 / 关心我	Selected to represent empathy directly
CAR2	Understanding	Does not understand how I feel / Understands how I feel	不理解我的感受 / 理解我的感受	Selected to represent affective understanding
CAR3	Understanding	Is insensitive to my circumstances / Is sensitive to my circumstances	不体察我的处境 / 体察我的处境	Selected to represent contextual understanding
CAR4	Responsiveness	Is unresponsive to my concerns / Is responsive to my concerns	不回应我的关切 / 回应我的关切	Selected to represent response to expressed concerns
CAR5	Responsiveness	Ignores my needs / Responds to my needs	忽视我的需要 / 回应我的需要	Selected to represent response to student needs
MK1	Marker	I prefer documents with balanced spacing.	我更喜欢间距均衡的文档。	Neutral preference
MK2	Marker	I like arranging objects in consistent patterns.	我喜欢按照一致的方式摆放物品。	Neutral preference
MK3	Marker	I prefer following the same daily routine when possible.	在条件允许时，我更喜欢遵循相同的日常安排。	Neutral preference
MK4	Marker	I notice when visual elements are unevenly aligned.	我会注意到视觉元素排列不整齐的情况。	Neutral preference

Supplementary Table S4. Development, translation, content validity, and EFA summary

Stage	Specification	Result or decision
Conceptual specification	Six dimensions mapped to Analects passages and Confucian educational interpretation	Responsive instruction explicitly treated as an interpretive synthesis of 11.22 and 6.21
Candidate pool	36 items; six per proposed dimension	All items fielded in Study 1
Expert panel	Seven experts: two Confucian philosophy, two educational psychology, two higher-education pedagogy, one psychometrics	Rated relevance, clarity, and source fidelity on four-point scales
Content validity	Item-level CVI range .86-1.00; scale-level average .95	Eleven items reworded before field administration
Translation	One bilingual educational-psychology researcher translated established measures	A second translator who had not seen the originals completed back-translation
Adjudication	Two educational psychologists and one bilingual higher-education researcher	Resolved translation discrepancies
Cognitive interviews	12 postsecondary students	Focused on comprehension, referent consistency, and culturally unnatural wording
Initial empirical screening	Primary loading, cross-loading, communality, loading separation, source fidelity, and content coverage	Normal criteria: primary loading >= .50; communality >= .40; largest cross-loading < .30; loading separation >= .20
Removal pattern	12 items removed	Six low primary loadings; five cross-loading or separation problems; one redundant item with weaker source fidelity
Final scale	24 items; four items per dimension	Maximum-likelihood EFA with promax rotation re-estimated on retained items

Factor	Items	Loading range	Eigenvalue	Variance %	Cumulative %	α	ω
F1 Benevolent concern	4	.75-.84	4.28	17.82	17.82	0.88	0.89
F2 Inclusive teaching	4	.69-.85	3.72	15.49	33.31	0.86	0.87
F3 Patient guidance	4	.79-.86	3.34	13.91	47.22	0.89	0.90
F4 Responsive instruction	4	.73-.78	2.96	12.34	59.56	0.85	0.86
F5 Norm-guided care	4	.66-.74	2.42	10.08	69.64	0.82	0.83
F6 Moral modeling	4	.72-.83	2.01	8.37	78.01	0.87	0.88

Note. Parallel analysis retained six factors. The sixth observed eigenvalue was 2.01 compared with a 95th-percentile random eigenvalue of .97; the seventh observed eigenvalue was .78 compared with a random eigenvalue of .91. The retained solution accounted for 78.01% of variance.

Supplementary Table S5. Sample characteristics and sequential screening flow

Category	Level	**Study 1** ($N = 326$)	**Study 2** ($N = 512$)
Sample size	Analytical sample	326	512
Gender	Female	178 (54.6%)	286 (55.9%)
Gender	Male	148 (45.4%)	226 (44.1%)
Grade	First year	88 (27.0%)	130 (25.4%)
Grade	Second year	91 (27.9%)	138 (27.0%)
Grade	Third year	83 (25.5%)	129 (25.2%)
Grade	Fourth year	64 (19.6%)	115 (22.5%)
Age	Mean (SD), years	20.12 (1.31)	20.18 (1.37)
Family SES	Mean (SD), 1-5 scale	3.09 (0.78)	3.13 (0.81)
Prior achievement	Self-reported GPA percentile, mean (SD)	67.4 (15.2)	68.1 (15.7)
Institution	Jianghan University (comprehensive)	194 (59.5%)	311 (60.7%)
Institution	Hubei University of Education (normal)	80 (24.5%)	126 (24.6%)
Institution	Hubei Urban Construction Vocational and Technical College (vocational)	52 (16.0%)	75 (14.6%)

Study	Stage	Value
Study 1	Field period	October-November 2025
Study 1	Opened survey	382
Study 1	Consented	361
Study 1	Submitted	352
Study 1	Excluded: more than 20% missing	8
Study 1	Excluded: failed either instructed-response attention check	7
Study 1	Excluded: at least 80% identical substantive responses	6
Study 1	Excluded: duration below 6.2 minutes	5
Study 1	Final analytical sample	326
Study 1	Retained percentage of submitted questionnaires	92.6%
Study 1	Item-level missingness after exclusions	0.8%
Study 2	Field period	March-April 2026
Study 2	Opened survey	604
Study 2	Consented	575
Study 2	Submitted	558
Study 2	Excluded: more than 20% missing	14
Study 2	Excluded: failed either instructed-response attention check	13
Study 2	Excluded: at least 80% identical substantive responses	11
Study 2	Excluded: duration below 6.0 minutes	8
Study 2	Final analytical sample	512
Study 2	Retained percentage of submitted questionnaires	91.8%
Study 2	Item-level missingness after exclusions	0.6%

Note. Eligibility required age 18 years or older, current enrollment at a participating institution, and at least four weeks of experience in the rated course. Study 2 used course sections that had not participated in Study 1. Participation was voluntary, anonymous, and uncompensated.

Supplementary Table S6. Measurement and structural model comparisons

Family	Model	χ^2	df	χ^2/df	CFI	TLI	RMSEA	SRMR	AIC	BIC	Interpretation
Measurement	Correlated six-factor PRBT	371.82	237	1.57	0.972	0.966	0.033	0.035			Clear and stable dimension interpretation
Measurement	Second-order PRBT	421.17	246	1.71	0.963	0.957	0.039	0.043			Adequate support for qualified total-score use
Measurement	Bifactor diagnostic	348.92	228	1.53	0.976	0.969	0.032	0.031			Best global fit; two specific factors had weak residual reliability
Measurement	Full measurement model	1548.39	833	1.86	0.957	0.949	0.041	0.039			Good fit
Measurement	One-factor model	6124.65	902	6.79	0.681	0.657	0.107	0.119			Poor fit
Measurement	PRBT and COT combined	2214.83	849	2.61	0.918	0.906	0.056	0.071			Inferior to treating the constructs as distinct
Measurement	COT five-item one-factor model	10.84	5	2.17	0.993	0.986	0.048	0.019			Adequate short-form benchmark
Structural	Direct effects only	1811.24	868	2.09	0.921	0.914	0.051	0.061	58126	58402	Inferior fit
Structural	TSRQ single mediator	1738.40	865	2.01	0.943	0.937	0.045	0.050	57910	58203	Improved over direct-effects model
Structural	BPNS single mediator	1722.91	865	1.99	0.946	0.940	0.044	0.049	57863	58156	Improved over direct-effects model
Structural	Parallel mediators	1664.83	861	1.93	0.954	0.947	0.043	0.044	57714	58033	Marginally strongest CFI and SRMR
Structural	Proposed serial model	1677.31	862	1.95	0.952	0.945	0.043	0.046	57702	58016	Lowest AIC and BIC; focal theoretically ordered model
Structural	Reverse serial model	1694.25	862	1.97	0.950	0.943	0.044	0.047	57829	58143	Less favorable information criteria

Supplementary Table S7. Reliability, descriptive statistics, correlations, and discriminant validity

Construct or dimension	Items	CR	AVE	α	ω	ω_h	ECV
PRBT total composite	24			0.93	0.94	0.81	0.64
Benevolent concern	4	0.85	0.60	0.84	0.85		
Inclusive teaching	4	0.88	0.65	0.87	0.88		
Patient guidance	4	0.87	0.63	0.86	0.87		
Responsive instruction	4	0.83	0.56	0.82	0.83		
Norm-guided care	4	0.85	0.60	0.84	0.85		
Moral modeling	4	0.80	0.50	0.80	0.81		
Care-oriented teaching	5	0.90	0.64	0.89	0.90		
Teacher-student relationship quality	5	0.88	0.58	0.87	0.88		
Basic psychological need satisfaction	5	0.87	0.57	0.86	0.87		
Learning motivation	5	0.88	0.59	0.87	0.88		
School belonging	5	0.89	0.61	0.88	0.89		
Academic engagement	5	0.90	0.64	0.89	0.90		

Variable	M	SD	PRBT	COT	TSRQ	BPNS	LM	SB	AE
PRBT	4.02	0.56	1.00	0.64	0.67	0.55	0.51	0.56	0.48
COT	3.91	0.61	0.64	1.00	0.61	0.48	0.43	0.50	0.41
TSRQ	3.86	0.62	0.67	0.61	1.00	0.58	0.53	0.60	0.46
BPNS	3.72	0.58	0.55	0.48	0.58	1.00	0.62	0.57	0.54
LM	3.78	0.57	0.51	0.43	0.53	0.62	1.00	0.55	0.65
SB	3.69	0.64	0.56	0.50	0.60	0.57	0.55	1.00	0.49
AE	3.63	0.60	0.48	0.41	0.46	0.54	0.65	0.49	1.00

Construct	PRBT	COT	TSRQ	BPNS	LM	SB	AE
PRBT	0.79						
COT	0.69	0.80					
TSRQ	0.76	0.68	0.76				
BPNS	0.62	0.55	0.66	0.75			
LM	0.60	0.47	0.58	0.72	0.77		
SB	0.63	0.57	0.67	0.65	0.64	0.78	
AE	0.58	0.51	0.52	0.63	0.72	0.55	0.80

Note. The HTMT table diagonal contains square roots of AVE. The PRBT–COT correlation was .64 and the corresponding HTMT value was .69. The largest HTMT value was .76.

Supplementary Table S8. Measurement invariance and multigroup structural stability

Grouping	Model	χ^2	df	CFI	TLI	RMSEA	SRMR	$\triangle$CFI	$\triangle$RMSEA	Decision
Gender	Configural	1134.52	660	0.953	0.945	0.040	0.044			Acceptable
Gender	Metric	1161.84	678	0.951	0.945	0.039	0.046	-0.002	-0.001	Supported
Gender	Scalar	1198.22	696	0.948	0.943	0.041	0.047	-0.003	0.002	Supported
Gender	Residual	1247.63	720	0.944	0.941	0.042	0.049	-0.004	0.001	Partial support
Grade: years 1-2 vs 3-4	Configural	1298.77	992	0.950	0.942	0.039	0.046			Acceptable
Grade: years 1-2 vs 3-4	Metric	1337.18	1016	0.948	0.942	0.039	0.047	-0.002	0.000	Supported
Grade: years 1-2 vs 3-4	Scalar	1385.40	1040	0.945	0.940	0.040	0.049	-0.003	0.001	Supported

Grouping	Constraint	ΔCFI	Interpretation
Gender	All focal structural paths constrained equal	-0.001	Broadly comparable
Grade: years 1-2 vs 3-4	All focal structural paths constrained equal	-0.001	Broadly comparable

Supplementary Table S9. Structural paths and bootstrap effects

Path	β	SE	z	p	95% CI L	95% CI U
PRBT -> TSRQ	0.56	0.041	13.66	$<$ .001	0.48	0.64
TSRQ -> BPNS	0.48	0.043	11.16	$<$ .001	0.40	0.56
TSRQ -> Learning motivation	0.18	0.052	3.46	.001	0.08	0.28
TSRQ -> School belonging	0.22	0.050	4.40	$<$ .001	0.12	0.32
BPNS -> Learning motivation	0.41	0.049	8.37	$<$ .001	0.32	0.50
BPNS -> School belonging	0.37	0.047	7.87	$<$ .001	0.28	0.46
PRBT -> Learning motivation	0.16	0.055	2.91	.004	0.05	0.27
PRBT -> School belonging	0.14	0.052	2.69	.007	0.04	0.24
COT -> Learning motivation	0.12	0.051	2.35	.019	0.02	0.22
COT -> School belonging	0.13	0.050	2.60	.010	0.03	0.23

Effect	Estimate	95% CI L	95% CI U	Hypothesis
PRBT -> TSRQ -> Learning motivation	0.101	0.047	0.169	H3
PRBT -> TSRQ -> School belonging	0.123	0.062	0.192	H3
PRBT -> TSRQ -> BPNS -> Learning motivation	0.110	0.063	0.168	H4
PRBT -> TSRQ -> BPNS -> School belonging	0.099	0.054	0.157	H4
Total indirect effect on learning motivation	0.211	0.142	0.289	H3–H4
Total indirect effect on school belonging	0.222	0.149	0.305	H3–H4
Total effect on learning motivation	0.371	0.286	0.461	H1
Total effect on school belonging	0.362	0.279	0.451	H2

Note. Confidence intervals are bias-corrected intervals from 5,000 case-level bootstrap resamples. The indirect effects are cross-sectional associations and do not establish temporal mediation.

Supplementary Table S10. Incremental validity

Outcome	Model	β COT	β PRBT	R^2	ΔR^2	f^2
Learning motivation	Step 1: covariates			0.118		
Learning motivation	Step 2: + COT	0.39		0.318	0.200	0.293
Learning motivation	Step 3: + PRBT	0.2	0.31	0.379	0.061	0.098
School belonging	Step 1: covariates			0.104		
School belonging	Step 2: + COT	0.44		0.354	0.250	0.387
School belonging	Step 3: + PRBT	0.22	0.34	0.423	0.069	0.120
Academic engagement	Step 1: covariates			0.089		
Academic engagement	Step 2: + COT	0.35		0.265	0.176	0.239
Academic engagement	Step 3: + PRBT	.19	0.29	0.318	0.053	0.078

Supplementary Table S11. Robustness and common-method sensitivity checks

Check	Target estimate	Estimate	SE	95% CI L	95% CI U	Interpretation
Primary SEM	PRBT -> TSRQ	0.560	0.041	0.480	0.640	Stable
Marker-variable model	PRBT -> TSRQ	0.540	0.043	0.460	0.620	Stable
Robust maximum likelihood	PRBT -> TSRQ	0.550	0.042	0.470	0.630	Stable
Excluding flagged careless responders	PRBT -> TSRQ	0.570	0.040	0.490	0.650	Stable
Controlling academic engagement	PRBT total effect on LM	0.350	0.047	0.260	0.440	Stable
Alternative outcome model	PRBT total effect on SB	0.340	0.048	0.250	0.430	Stable
ULMC comparison	Common method factor variance	0.180				Descriptive sensitivity analysis
ULMC path stability	Maximum absolute change in focal standardized paths	0.020				No focal path changed by more than .02
Harman single-factor check	First unrotated factor variance	0.274				Does not rule out method bias
Marker correlations	Range with focal constructs	-.06 to .08				Neutral marker showed small correlations

Supplementary Table S12. Construct-level covariance matrix

Variable	PRBT	COT	TSRQ	BPNS	LM	SB	AE
PRBT	0.3136	0.2186	0.2326	0.1786	0.1628	0.2007	0.1613
COT	0.2186	0.3721	0.2307	0.1698	0.1495	0.1952	0.1501
TSRQ	0.2326	0.2307	0.3844	0.2086	0.1873	0.2381	0.1711
BPNS	0.1786	0.1698	0.2086	0.3364	0.2050	0.2116	0.1879
LM	0.1628	0.1495	0.1873	0.2050	0.3249	0.2006	0.2223
SB	0.2007	0.1952	0.2381	0.2116	0.2006	0.4096	0.1882
AE	0.1613	0.1501	0.1711	0.1879	0.2223	0.1882	0.3600

Note. This matrix is the construct-level covariance matrix obtained from the Study 2 standard deviations and Pearson correlations reported in Supplementary Table S7. The participant-level item covariance matrix is not included.

Data availability

The participant-level survey data are not deposited in a public repository because the consent materials and institutional ethics conditions did not authorize unrestricted public release. This supplementary package includes the item dictionary, construct-level correlation and covariance matrices, variable codebook, and analysis syntax. Additional de-identified derived outputs may be made available by the corresponding author upon reasonable request, subject to ethics approval and confirmation that the proposed use is consistent with participant consent.

Source measures

The comparison and criterion measures were based on Teven and McCroskey's Perceived Caring Scale; Ang's Teacher–Student Relationship Inventory and the student version reported by Ang, Ong, and Li; Chen and colleagues' Basic Psychological Need Satisfaction and Frustration Scale; Vallerand and colleagues' Academic Motivation Scale; Goodenow's Psychological Sense of School Membership scale; and Schaufeli and colleagues' student engagement measure. Full bibliographic information appears in the main manuscript.
